# Genomes and secretomes of Ascomycota fungi reveal diverse functions in plant biomass decomposition and pathogenesis

**DOI:** 10.1186/s12864-019-6358-x

**Published:** 2019-12-12

**Authors:** Jean F. Challacombe, Cedar N. Hesse, Lisa M. Bramer, Lee Ann McCue, Mary Lipton, Samuel Purvine, Carrie Nicora, La Verne Gallegos-Graves, Andrea Porras-Alfaro, Cheryl R. Kuske

**Affiliations:** 1 Bioscience Division, Los Alamos National Laboratory, Los Alamos, NM 87545 USA; 2 Present address: Colorado State University, College of Agricultural Sciences, 301 University Ave, Fort Collins, CO 80523 USA; 3 Horticultural Crops Research, USDA ARS, Corvallis, OR USA; 4 Applied Statistics & Computational Modeling, Pacific Northwest National Laboratory, Richland, Washington, USA; 5 Biological Sciences Division, Pacific Northwest National Laboratory, Richland, Washington, 99352 USA; 6 Department of Biology, Western Illinois University, Macomb, IL 61455 USA

**Keywords:** Ascomycota, Fungi, Arid, Grassland, Soil, Biocrust, Genome, Secretome, Lifestyle, Plants

## Abstract

**Background:**

The dominant fungi in arid grasslands and shrublands are members of the Ascomycota phylum. Ascomycota fungi are important drivers in carbon and nitrogen cycling in arid ecosystems. These fungi play roles in soil stability, plant biomass decomposition, and endophytic interactions with plants. They may also form symbiotic associations with biocrust components or be latent saprotrophs or pathogens that live on plant tissues. However, their functional potential in arid soils, where organic matter, nutrients and water are very low or only periodically available, is poorly characterized.

**Results:**

Five Ascomycota fungi were isolated from different soil crust microhabitats and rhizosphere soils around the native bunchgrass *Pleuraphis jamesii* in an arid grassland near Moab, UT, USA. Putative genera were *Coniochaeta*, isolated from lichen biocrust, *Embellisia* from cyanobacteria biocrust*, Chaetomium* from below lichen biocrust, *Phoma* from a moss microhabitat, and *Aspergillus* from the soil. The fungi were grown in replicate cultures on different carbon sources (chitin, native bunchgrass or pine wood) relevant to plant biomass and soil carbon sources. Secretomes produced by the fungi on each substrate were characterized. Results demonstrate that these fungi likely interact with primary producers (biocrust or plants) by secreting a wide range of proteins that facilitate symbiotic associations. Each of the fungal isolates secreted enzymes that degrade plant biomass, small secreted effector proteins, and proteins involved in either beneficial plant interactions or virulence. *Aspergillus* and *Phoma* expressed more plant biomass degrading enzymes when grown in grass- and pine-containing cultures than in chitin. *Coniochaeta* and *Embellisia* expressed similar numbers of these enzymes under all conditions, while *Chaetomium* secreted more of these enzymes in grass-containing cultures.

**Conclusions:**

This study of Ascomycota genomes and secretomes provides important insights about the lifestyles and the roles that Ascomycota fungi likely play in arid grassland, ecosystems. However, the exact nature of those interactions, whether any or all of the isolates are true endophytes, latent saprotrophs or opportunistic phytopathogens, will be the topic of future studies.

## Background

In arid grasslands and shrublands, the dominant fungi in surface soils are members of the Ascomycota phylum [1, 2]. In contrast to higher organic-matter forest soils, where Basidiomycota fungi are the dominant biomass, the Ascomycota are important drivers in carbon and nitrogen cycling [3–5] and plant interactions [6]. However, their functions in arid soils, where organic matter, nutrients and water are very low or only periodically available, are poorly characterized. Potential roles include soil stability against erosion, seasonal plant biomass decomposition, direct interactions with plants as endophytes or as pathogens that induce selective disassembly of plant tissues. Recent work shows that these soil fungi are integral members of cyanobacteria-dominated biological soil crusts and belowground microhabitats, where they may facilitate transport of nutrients acting as mycorrhizae and promote plant growth and survival and contribute to biocrust stability. The most abundant fungal genera in arid soil biocrusts and rhizospheres include *Aspergillus, Alternaria, Acremonium, Chaetomium, Cladosporium, Coniochaeta, Fusarium, Mortierella, Preussia, Phoma* and *Rhizopus* [1, 7, 8] (Ndinga Muniania et al. 2019, in review).

We examined the genomes and secreted proteomes from five Ascomycota genera that were abundant in multiple arid land microhabitats (Ndinga Muniania et al. 2019, in review) [7–9]. These isolates from the arid grassland biome represent ecologically enigmatic members of the orders Pleosporales and Sordariales, which are found in high abundance associated with biological soil crusts and in plant root zones (Ndinga Muniania et al. 2019, in review) [2]. Although some members of our proposed genera have been hypothesized to be root-associated endophytes, all display some degree of saprophytic ability and may have the capability to decompose cellulose or other plant-derived carbohydrates. These five fungi were grown in replicate cultures with three different carbon sources including sawdust of *Pinus teada* (pine), and an arid land bunchgrass *Hilaria jamesii* (*Pleuraphis jamesii*, James’ Galleta), as well as powdered chitin; all of these substrates are relevant to plant biomass decomposition and fungal growth in temperate soils. The genomes were sequenced and the secreted proteomes of the five fungi (secretomes) were identified and compared, revealing a diverse range in the expression of proteins involved in fungal metabolism, growth, secondary metabolite production and virulence.

Visual examination of the fungal cultures revealed melanized structures, a common characteristic of dark septate fungal species. Dark septate fungi (DSF) play many roles in soil systems, contributing to soil nutrient cycling, soil stabilization, and plant survival [2, 10, 11], but the precise roles of individual DSF, their distribution, and diversity in soil systems are still poorly understood. There is evidence that DSF play an important role in plant survival in arid grasslands [1, 2, 12]. The protective melanin pigment and resistant spores that allow survival in harsh conditions provide a competitive advantage to DSF with respect to other fungal taxa considering the increased temperature, solar radiation and xeric conditions that prevail in arid and semiarid soil environments. Our comparative genomic analyses showed that all of the fungi had the genetic capability to produce at least two types of melanin. Our results also demonstrated protein signatures characteristic of fungal growth on different carbon substrates, including multiple expressed carbohydrate active enzymes (CAZymes) involved in the decomposition of plant biomass. The expression of proteins involved in various metabolic pathways, mitosis and meiosis, signaling, vesicular transport, and chitin metabolism suggested that the fungi were growing actively in the cultures, although there were some differences across the five fungal genera and among the three different substrates.

The expression of small secreted proteins, secondary metabolite anchor genes, siderophore biosynthesis genes, and other functional categories related to pathogenesis and defense, particularly in *Embellisia*, *Chaetomium* and *Phoma*, suggested wide ecological niches and functional plasticity for these Ascomycota isolates including known saprotrophic and possibly virulent capabilities toward plants, with all of them likely to participate in some type of symbiotic interaction with plants. One of the isolates, an *Aspergillus* that was most closely related to *A. fumigatus* via genome comparisons, is a commonly isolated fungus in this system but is not considered a true DSF. The insights that we gained through comparisons of the genomes and secretomes of the Ascomycota isolates will advance our fundamental knowledge of the functional roles and ecological adaptations that Ascomycota DSF have in arid soil microbial communities.

## Results

This study compared the genomes and secretomes of five fungal genera in the Ascomycota phylum, following growth in culture in the presence of three different complex carbon sources (chitin, native bunchgrass or pine sawdust, 1% w/v in 0.2% sucrose), as well as 0.2% sucrose alone as a control. Chitin, *Hilaria jamesii* bunchgrass (cellulosic) and pine (lignocellulosic) are common carbon sources in temperate soils in the U.S. To assess the functional capabilities of the fungi, we compared the genomes and secretomes using a variety of bioinformatic approaches. For the secretome analyses, protein expression in the presence of each substrate was compared to protein expression in sucrose as the control.

### Genome sequencing, assembly and annotation statistics

Table 1 lists the sequencing, assembly and annotation statistics.

**Table 1:** Genome Sequencing, Assembly and Annotation Statistics

Genome	Median Coverage	N50	Max contig length	Total bases	Contigs	Coding Sequences
*Aspergillus* CK392 (FGC_1)	61.35	370,614	937,006	27,610,920	356	8810
*Coniochaeta* CK134 (FGC_2)	37.71	258,339	888,870	37,872,879	1013	10,628
*Embellisia* CK46 (FGC_3)	37.76	359,781	950,064	36,024,182	2580	12,047
*Chaetomium* CK152 (FGC_4)	31.5	38,802	179,509	34,976,647	3917	11,804
*Phoma* CK108 (FGC_5)	37.22	166,777	666,689	35,585,417	2526	10,223

SPOCS clique analysis identified 2632 proteins with homologs in all five genomes (Additional file 1)

### Secretome analysis

**Fig. 1:**
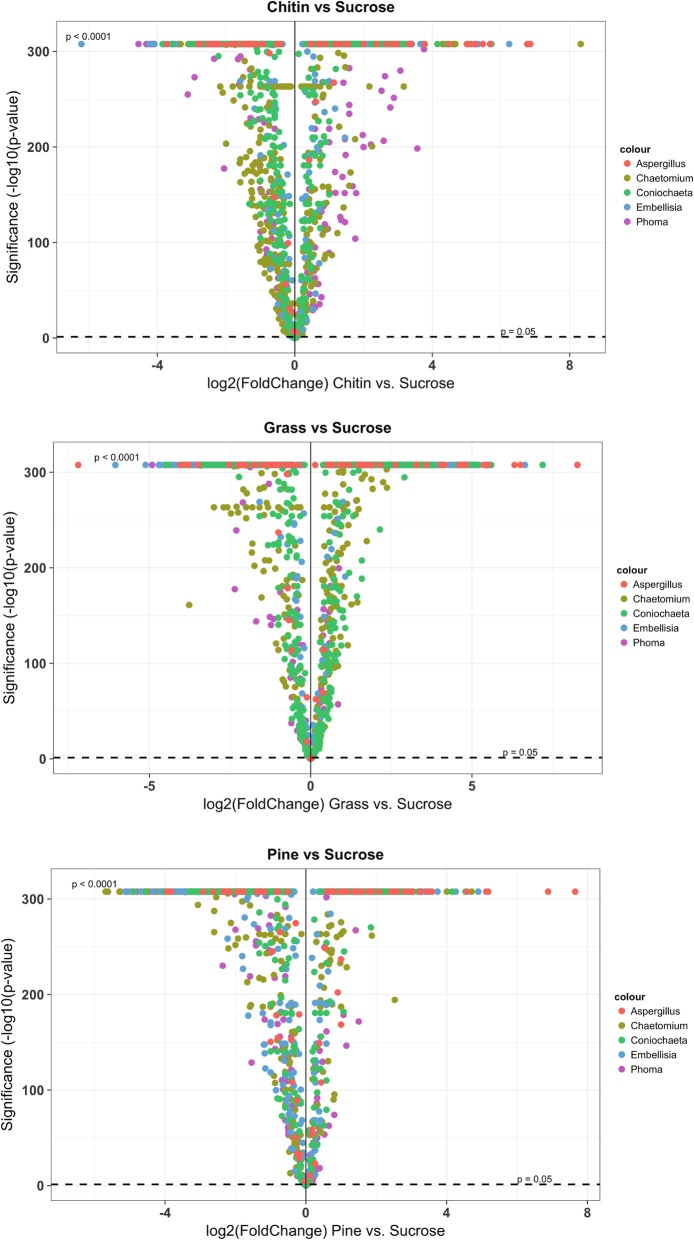
Volcano plots showing the fold change in protein expression of each fungus grouped by treatment (chitin, grass, pine) compared to the sucrose control. Dots represent individual proteins. On the x-axis is the log2(Fold Change) of the protein in each treatment compared to sucrose control. The y-axis shows the significance of the fold change as -log10(*p*-value) of the treatment compared to the sucrose control. Detailed information on how these values were obtained is presented in the methods section. The data used to generate this figure are from Additional file 3

**Fig. 2:**
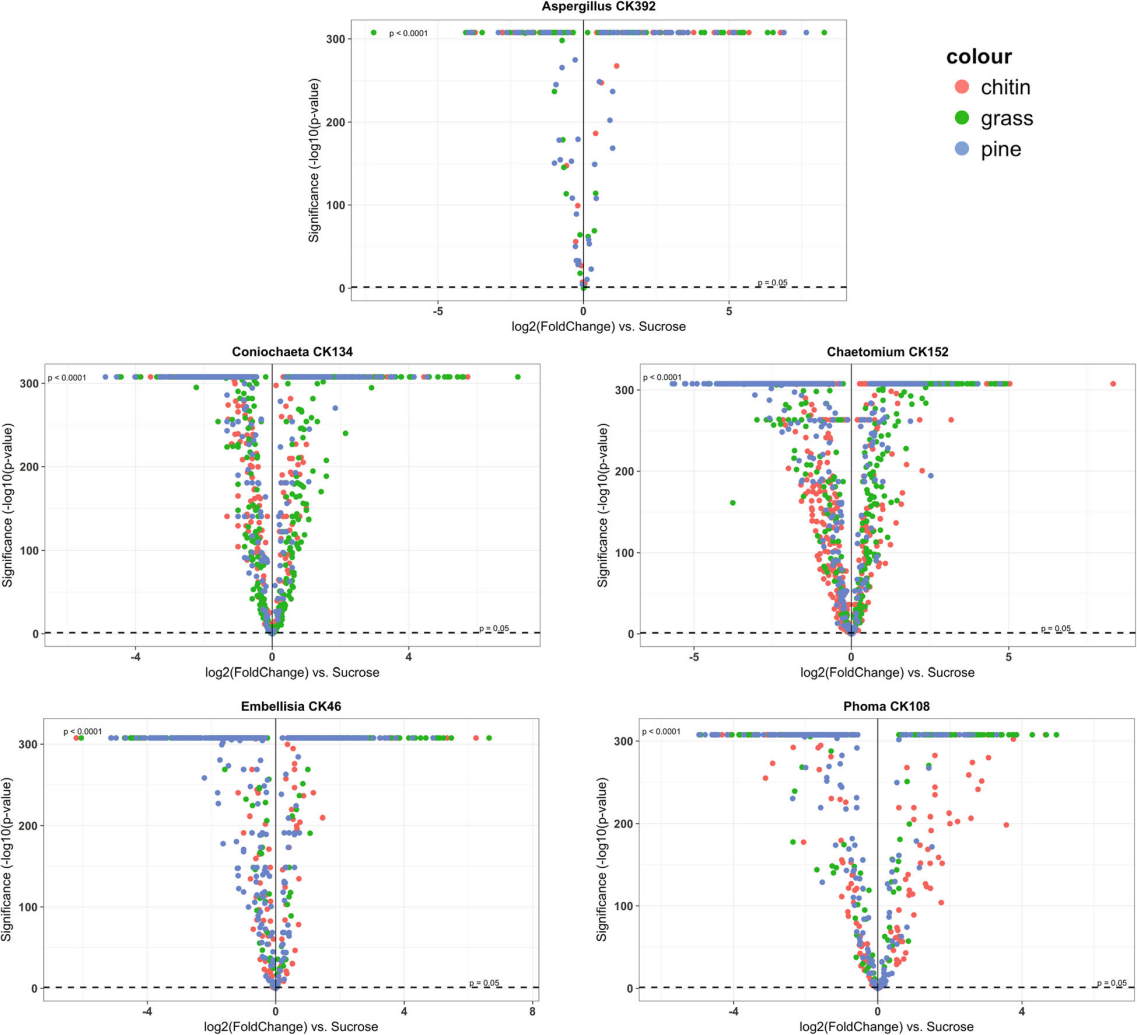
Volcano plots comparing the fold change in protein expression of each treatment, grouped by fungus. Dots represent individual proteins. On the x-axis is the log2(Fold Change) of the protein in each treatment compared to sucrose control. The y-axis shows the significance of the fold change as -log10(*p*-value) of the treatment compared to the sucrose control. Detailed information on how these values were obtained is presented in the methods section. The data used to generate this figure are from Additional file 3

The complete data sets of protein abundances for each fungus under each growth condition are in Additional file 2. Statistics and annotations for the proteins that were expressed in each growth condition are given in Additional file 3. The volcano plots in Figs. 1 and 2 show the protein expression patterns in the fungi during growth in chitin, grass and pine cultures. These plots were created from the data in Additional file 3. In Fig. 1, the data are grouped by culture condition (treatment), to facilitate comparison of the protein expression patterns in all of the fungi under each of the three culture conditions. In Fig. 2, there is one volcano plot for each fungus, to enable comparison of the protein expression patterns that occurred during growth of that fungus in each culture condition. Figures 1 and 2 illustrate the expression patterns of individual proteins, and the Figures in Additional files 4, 5, 6, 7, 8, 9, 10 and 11 show each of the volcano plots with all of the proteins labeled. While the plots and labels are small, zooming into regions of interest in these high-resolution figures shows the expression patterns of individual proteins of interest. The protein labels and corresponding annotations are listed in Additional file 3. In all of the volcano plots, the most highly significant values align at the top of the plots, with a maximum value of 307.698970004336, which represents (−log10(*p*-value of 2e-308); this is due to R’s representation of floating-point numbers by IEEE 754 64-bit binary numbers. The lowest non-zero *p*-value that can be represented is 2e-308, so numbers with absolute magnitude below this are treated as zero by R, and the maximum value at the top of the volcano plots is -log10(2e-308), or 307.698970004336. These are the most significant values.

Seven hundred thirty-five proteins had homologs in all five fungi and showed a change in expression in at least one fungus under at least one of the three conditions (Additional file 12). To better compare the expression of these proteins in the fungi under the different conditions, proteins were grouped by pathway membership (Additional file 12 ‘common pathways’ tab). The bar plots in Additional file 13 were generated from the data in Additional file 12 (‘common pathways’ tab) to illustrate the similarities and differences in the expression of protein components of metabolic pathways and other functional categories across the fungal isolates. These plots show trends in protein expression in all of the fungi under the different culture conditions (chitin, grass or pine biomass). For example, proteins with potential functions in fungal growth and metabolism (‘Amino sugar and nucleotide sugar metabolism’, ‘Cysteine and methionine metabolism’, ‘Lysine metabolism’, ‘Valine, leucine and isoleucine metabolism’) showed higher expression in *Chaetomium* CK152 when the fungus was grown in grass and chitin, but not as much when grown in pine. Only *Chaetomium* and *Coniochaeta* showed increased expression of proteins in the ‘Amino sugar and nucleotide sugar metabolism’ category. All of the fungi except *Aspergillus* showed increased expression of proteins in the ‘Purine and pyrimidine metabolism’, ‘Cysteine and methionine metabolism’ and ‘Calcium binding’ categories under all three conditions, and ‘Lysine metabolism’ under all conditions, except *Phoma*, which only expressed proteins in this category when grown in grass. Proteins involved in ‘Valine, leucine and isoleucine metabolism’ were expressed in all but *Aspergillus* under at least one condition. From the expression patterns in Figs. 1, 2 and the Figure in Additional file 13, along with the numbers reported in Table 2, *Coniochaeta* and *Chaetomium* expressed higher numbers of proteins when grown in the presence of chitin and grass, compared to growth in the presence of pine. However, there were some categories of proteins that were expressed in these two fungi under all three conditions, such as ‘Plant polysaccharide degradation’, ‘Amino acid metabolism’, ‘Antioxidant’, ‘Benzoate degradation’, ‘Chromatin structure and function’, ‘Cytoskeleton’, ‘Glycolysis/gluconeogenesis’, ‘L-serine biosynthesis’, ‘Lysine metabolism’, ‘Nitrogen metabolism’, ‘Oxidative phosphorylation’, ‘Pathogenesis’, ‘Pentose phosphate pathway’, indicating that these two fungi are more similar to each other among the five fungi included in this study.

**Table 2:** Number of proteins that showed increased expression (fold change) under each condition compared to sucrose control

Genome	Number of proteins with fold change > 0 under any condition compared to sucrose (% of total CDS)	Number of proteins with fold change > 0 when grown in chitin vs sucrose (% of total CDS)	Number of proteins with fold change > 0 when grown in grass vs sucrose (% of total CDS)	Number of proteins with fold change > 0 when grown in pine vs sucrose (% of total CDS)	Number of proteins with fold change > 0 under all three conditions compared to sucrose (% of total CDS)
*Aspergillus* CK392 (FGC_1)	315(3.6%)	104(1.2%)	101(1.2%)	110(1.3%)	72(0.8%)
*Coniochaeta* CK134 (FGC_2)	2275(21.4%)	809(7.6%)	876(8.2%)	590(5.6%)	481(4.5%)
*Embellisia* CK46 (FGC_3)	1504(12.5%)	631(5.2%)	347(2.9%)	526(4.4%)	246(2.0%)
*Chaetomium* CK152 (FGC_4)	2306(19.5%)	1050(8.9%)	731(6.2%)	5254.5%)	398(3.4%)
*Phoma* CK108 (FGC_5)	975(9.5%)	307(3.0%)	318(3.1%)	350(3.4%)	148(1.5%)

Data for this table were compiled from Additional file 3. CDS: coding sequences

*Aspergillus* and *Phoma* had similar numbers of proteins with increased expression on all three substrates (Table 2) but showed some differences in functional categories of proteins that were expressed during growth on the different carbon substrates (Additional file 13). *Phoma* showed notably increased expression of proteins involved in ‘Starch and sucrose metabolism’ and ‘Calcium binding’ proteins when grown in grass, and in ‘Transport’, ‘Signaling’, ‘Siderophore biosynthesis’, ‘Lipid metabolism’, ‘Glycolysis/glyconeogenesis’, ‘Glycolipid transfer’, ‘Calcium binding’, ‘Antioxidant’, ‘Aminoacyl-tRNA biosynthesis’, and ‘Amino acid metabolism’ categories when grown in chitin. In pine, *Phoma* showed the highest protein expression in the ‘Transport’, ‘Starch and sucrose metabolism’, ‘Signaling’, ‘Siderophore biosynthesis’, ‘Pathogenesis’, ‘Nitrogen metabolism’, ‘Lipid metabolism’, and ‘Mitosis and meiosis’ categories. *Phoma* also showed the lowest overall protein expression in pine compared to the other substrates.

As shown in Fig. 1, *Aspergillus* had very highly significant protein expression values on all three substrates (red dots along the top of the plots, which align at the limit of R’s ability to represent very small *p*-values). This may reflect fast growth on the substrates, and the production of a lot of mycelium in a very short period of time. This explanation is supported by the large expression of cytoskeletal proteins in *Aspergillus* when grown in pine, as shown in Additional file 13. However, *Aspergillus* notably showed an overall lower number of proteins expressed under any condition (Additional file 12 (‘common pathways’ tab) and Additional file 13.

*Embellisia* had increased protein expression in the categories of ‘Amino acid metabolism’, ‘Aminoacyl-tRNA biosynthesis’, ‘Antioxidant’, ‘Calcium binding’, ‘Cell wall organization’, ‘Cysteine and methionine metabolism’, ‘Cytoskeleton’, ‘Fatty acid metabolism’, ‘Glycerophospholipid metabolism’, ‘Glycolipid transfer’, ‘Glycolysis/gluconeogenesis’, ‘Lipid metabolism’, ‘Lysine metabolism’, ‘Mitochondrial protein import’, ‘NO detoxification’, ‘Oxidative phosphorylation’, ‘Pathogenesis’, ‘Pentose phosphate pathway’, ‘Plant polysaccharide degradation’, ‘Stress response’, ‘Starch and sucrose metabolism’, ‘Signaling’, ‘Siderophore biosynthesis’ when grown on all three substrates (chitin, grass and pine). A few categories typically associated with housekeeping functions, showed increased protein expression in all of the fungi under most or all of the culture conditions: ‘Protein folding, sorting and degradation’, ‘Protein processing’, and ‘Cell wall organization’.

### Pathway analysis

**Fig. 3:**
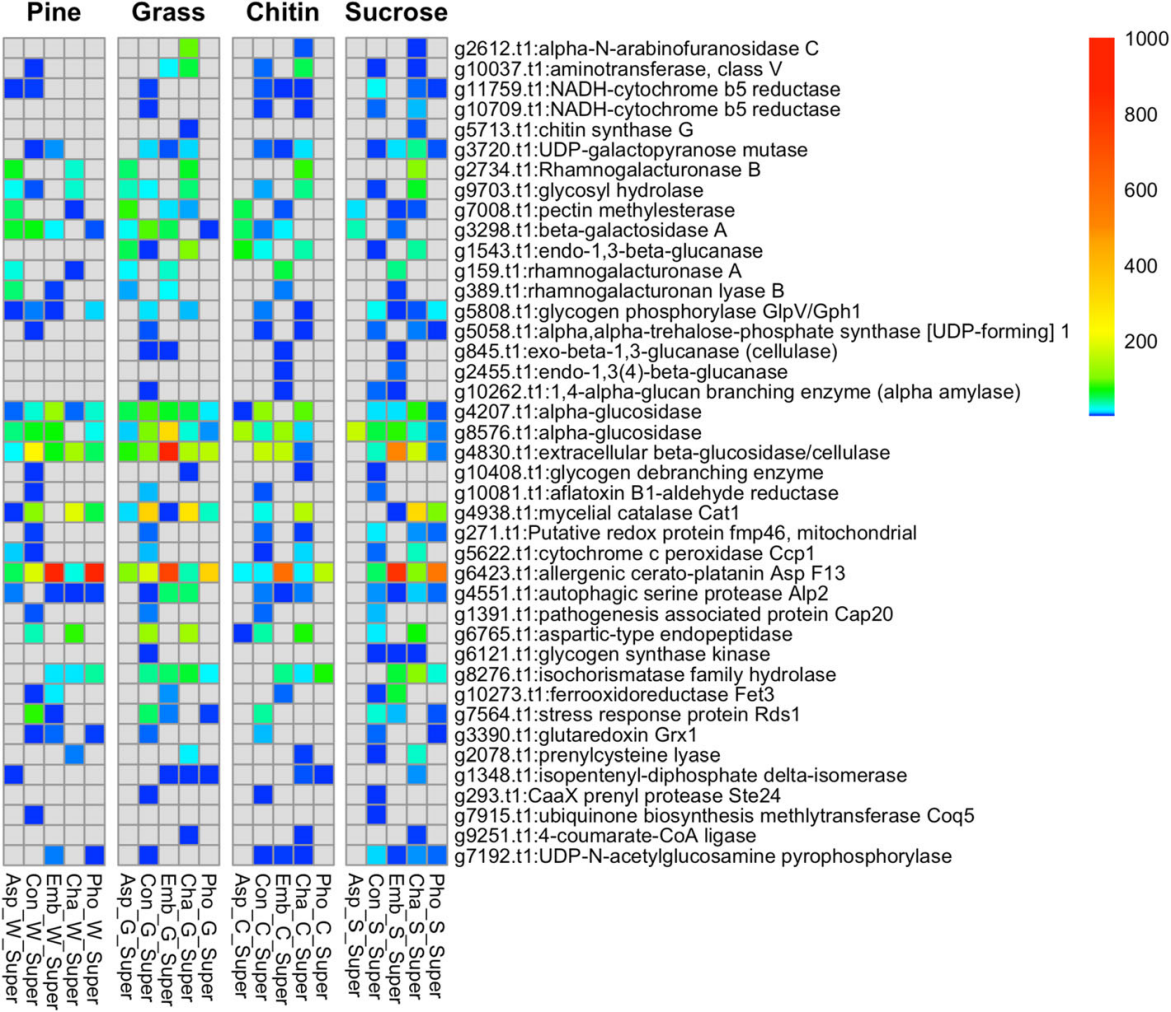
Heatmap showing the expression levels of proteins with annotated functions in pathways for plant biomass degradation, defense and virulence (pathogenesis). Total protein counts in pooled samples (from combined replicates) for each treatment condition are shown for each fungus. The data used to generate this figure are from Additional file 14

Overall trends in the expression of pathway components are apparent in the Figure Additional file 13, and it is clear that there are differences in protein expression among the fungi with respect to the carbon substrates. However, to better evaluate the expressed proteins with respect to fungal functions and lifestyles, we focused on the pathways involved in the degradation of lignocellulosic plant materials, such as cellulose, pectin, lignin and hemicellulose, as these may provide clues about the lifestyles of these fungi. While all of the candidate DSF isolates are likely saprobes that utilize plant biomass from decaying wood, leaves and litter, they could also be phytopathogens. *Embellisia* and *Phoma* are members of larger fungal groups that include plant pathogens. *Embellisia* is most closely related to *Alternaria* [13], a genus that contains many known plant pathogens [14, 15], and *Phoma* is part of a complex with *Leptosphaerulina* and other genera that include plant pathogens [16–18]. To gain evidence for potential phytopathogenicity, we included proteins with functions in defense and pathogenesis in the targeted comparative analyses. The heatmaps in Fig. 3 were generated from pooled sample data (columns C-G) of Additional file 2, filtered to include only the proteins with homologs in all five fungal genomes and only the pathways involved in plant biomass decomposition, defense and pathogenesis (Additional file 12 (‘selected pathways’ tab)). Data used to create the heatmaps is given in Additional file 14. Heatmaps showing all of the replicates for each treatment are shown in Additional file 15.

The heatmaps in Fig. 3 and Additional file 15 show that only three proteins, all with annotated functions indicating that they are involved in plant biomass degradation, were expressed when *Aspergillus* was grown in sucrose: pectin methylesterase (*Aspergillus* protein ID g4042.t1, *Chaetomium* ID g7008.t1 in heatmap), beta-galactosidase A (*Aspergillus* g5886.t1/*Chaetomium* g3298.t1) and alpha-glucosidase (*Aspergillus* g6893.t/ *Chaetomium* g8576.t1). These three proteins were also expressed by *Aspergillus* in the other conditions (chitin, grass, pine). The pectin methylesterase was not expressed in *Coniochaeta* or *Phoma* under any condition but was expressed by *Embellisia* at low levels in sucrose, chitin and grass cultures, while *Chaetomium* expressed it at low levels when grown in sucrose, grass and pine. Pectin methylesterases degrade the pectin components in plant cell walls [19]. The beta-galactosidase A was not expressed by *Chaetomium* under any culture conditions, while it was expressed by *Embellisia* under all conditions, and in *Coniochaeta* when grown in chitin, grass and pine, but only in *Phoma* grown in grass and pine. Beta-galactosidases act on the xyloglucan components of plant cell walls [20]. Two additional proteins likely involved in plant biomass degradation were expressed by *Aspergillus* when grown in chitin- and grass-containing media: endo-1,3-beta-glucanase (*Aspergillus* g1472.t1/*Chaetomium* g1543.t1) and two alpha glucosidases (*Aspergillus* g5811.t1/*Chaetomium* g4207.t1; *Aspergillus* g6893.t1/*Chaetomium* g8576.t1); the alpha glucosidases were also expressed by *Aspergillus* grown in pine, and one of them was expressed by *Aspergillus* grown in sucrose, as well as *Coniochaeta* and *Embellisia* under all conditions, and *Chaetomium* in all conditions except pine; *Phoma* expressed it in all conditions except chitin. Alpha glucosidases degrade plant cell wall cellulose, among other plant-derived substrates [20, 21]. The endo-1,3-beta-glucanase was also expressed in *Coniochaeta* (sucrose, chitin) and *Chaetomium* (sucrose, chitin, grass). Endo-1,3-beta-glucanases can degrade cellulose, hemicellulose, lichenin, and beta-D-glucans in plant cell walls (https://brenda-enzymes.org/enzyme.php?ecno=3.2.1.6). Other notable proteins likely involved in plant biomass degradation, that were expressed differentially among the fungi included UDP-galactopyranose mutase (*Chaetomium* g3720.t1), a component of galactose metabolism and cell wall biosynthesis, with potential roles in pathogenesis [22]. This protein was expressed by *Coniochaeta* and *Embellisia* under all conditions, in *Chaetomium* (sucrose, chitin, grass), and *Phoma* expressed it only when grown in sucrose. A rhamnogalacturonase B (also called rhamnogalacturonan lyase B; *Chaetomium* g2734.t1) was expressed in *Aspergillus* grown in grass and pine, and in *Chaetomium* under all conditions. Another rhamnogalacturonan lyase B (*Chaetomium* g389.t1) was expressed in *Embellisia* under all conditions but was only expressed in *Aspergillus* when grown in grass and pine and was not expressed in the other three fungi under any condition. Rhamnogalacturonan lyases degrade rhamnogalacturonans, which are pectin-containing polysaccharide components of plant cell walls [20, 21].

Some proteins with annotated functions in plant biomass degradation and pathogenesis were expressed only in *Chaetomium*. One of these, alpha-N-arabinofuranosidase C (g2612.t1), functions in the degradation of arabinoxylan, a component of plant hemicellulose, and is also required for full virulence of rice blast fungus *Magnaporthe oryzae* [23]. Chitin synthase G (g5713.t1), also expressed by *Chaetomium*, may play a role in pathogenic plant interactions, as chitin synthesis plays a role in the virulence of the plant fungal pathogens *Botrytis cinerea* [24, 25], *Magnaporthe oryzae* [26], *Fusarium oxysporum* [27], *Fusarium verticillioides* [28], *Fusarium asiaticum* [29], *Gibberella zeae* [30], *Colletotrichum graminicola* [31] and *Ustilago maydis* [32, 33].

Other proteins with potential roles in plant pathogenicity and biomass degradation were expressed in both *Chaetomium* and *Coniochaeta*. These proteins included aminotransferase, class V (g10037.t1), NADH-cytochrome b5 reductase (g10709.t1), alpha,alpha-trehalose-phosphate synthase [UDP-forming] 1 (*Chaetomium* g5058.t1), and a glycogen debranching enzyme (*Chaetomium* g10408.t1). Aminotransferases enable fungi to acquire nutrients required for pathogenicity [34]. Cytochrome b5 reductase has been implicated in the virulence of phytopathogenic fungus *Zymoseptoria tritici* [35]. Trehalose is a potential source of carbon and may also protect proteins and membranes from external stressors, such as dehydration, heat, cold, and oxidation [36]. Glycogen debranching enzyme plays an important role in the metabolism of glycogen [37].

An extracellular beta-glucosidase/cellulase (*Chaetomium* 4830.t1) was expressed by *Coniochaeta*, *Embellisia* and *Chaetomium* under all conditions. Significantly, *Embellisia* had a very high expression of this protein when grown in the presence of grass. *Aspergillus* expressed this protein when grown in grass and pine, and *Phoma* expressed it when grown in all but chitin. Beta-glucosidase enzymes are involved in cellulose degradation, hydrolyzing cellobiose into glucose [38]. As key enzymes in the hydrolysis of cellulosic biomass, beta-glucosidases reduce cellobiose accumulation, relieving cellobiose-mediated feedback inhibition of cellobiohydrolases [39].

In the pathogenesis category, *Coniochaeta, Embellisia*, *Chaetomium*, and *Phoma* expressed an allergenic cerato-platanin Asp F13 (*Aspergillus* g2965.t1/*Chaetomium* g6423.t1) when grown under all conditions; *Aspergillus* did not express this protein when grown in sucrose but did express it under the other conditions. *Phoma* and *Embellisia* had the highest expression of this protein on all substrates. Cerato-platanins appear to play a role during fungus-plant interactions and may reduce the force needed to break the plant cell walls, aiding the penetration of plant cell walls by fungal hyphae [40]. Cerato-platinins also bind to chitin and may have an expansin-like function acting non-hydrolytically on cellulosic materials [41]. An aspartic-type endopeptidase (*Chaetomium* g6765.t1) was expressed by *Coniochaeta* and *Chaetomium* on all substrates, and by *Aspergillus* grown in chitin. This protein may be involved in both nutrition and pathogenesis [42]. *Embellisia*, *Chaetomium* and *Phoma* expressed an isochorismatase family hydrolase (*Chaetomium* g8276.t1), which is involved in siderophore biosynthesis, and this protein was also expressed in *Coniochaeta* when grown in grass.

While looking at differences in the expression of proteins that are present in all five fungi is informative, proteins that are uniquely present in each fungus may provide more specific clues about their lifestyles under each growth condition. Additional file 16 lists the proteins that were uniquely encoded in each fungal genome (not present in any of the others). The percentages of unique protein coding sequences in each fungal genome were 30.7% (*Aspergillus* CK392), 32.2% (*Coniochaeta* CK134 and *Embellisia* CK46), 39.4% (*Chaetomium* CK152) and 26.3% (*Phoma* CK108). The unique protein sets included a wide range of functions. For each fungus, a small number of the total set showed a fold change in expression under any of the culture conditions compared to the sucrose control. These numbers are indicated at the bottom of each sheet in Additional file 16. Annotated functions of these proteins included plant polysaccharide degradation, defense and pathogenesis, metabolism, cell wall related functions, and the cytoskeleton. Some of the proteins that showed increased expression under at least one condition fit the criteria of small secreted proteins (SSPs), which are defined below.

### Secondary metabolites

Soil fungi produce a wide range of natural products, which may be of medical, industrial and/or agricultural importance. Some of the natural products produced by fungi are toxins [43, 44], which can cause disease in plants and animals, while others are beneficial to humans (e.g., antibiotics [45, 46]). Certain fungal genera produce natural products (also called secondary metabolites) that are characteristic of their genus and/or species [47–50]. To examine the complement of genes involved in secondary metabolite biosynthesis, which may provide clues about the lifestyles of the Ascomycete fungi, secondary metabolite anchor genes (or backbone genes) were predicted in each fungal genome sequence using the SMIPS program [51]. We tried using anti-SMASH [52], which is the standard tool for this task, but many of the predicted fungal coding sequences were too small for it to produce complete results. The categories of enzymes identified by SMIPS may play roles in synthesizing secondary metabolites. The SMIPS predictions are based on protein domain annotations obtained by InterProScan [53]. Secondary metabolite (SM) anchor genes identified by SMIPS include polyketide synthases (PKS), non-ribosomal peptide synthetases (NRPS) and dimethylallyltryptophan synthase (DMATS). Table 3 lists the numbers of each of these anchor gene types, predicted by SMIPS in each fungal genome. The detailed SMIPS outputs are shown in Additional file 17.

**Table 3:** Number of secondary metabolite anchor genes and types predicted by the SMIPS program

Genome	FGC_1 *Aspergillus* CK392	FGC_2 *Coniochaeta* CK134	FGC_3 *Embellisia* CK46	FGC_4 *Chaetomium* CK152	FGC_5 *Phoma* CK108
NRPS genes	44	36	20	27	14
DMATS	15	3	5	4	6
NRPS	14	8	5	5	3
NRPS-PKS hybrid	2	5	1	0	0
PKS	13	20	9	18	5
NRPS- and PKS-like genes	15	11	10	10	11
NRPS-like	8	5	9	7	4
PKS-like	7	6	1	3	7
Single domain genes	16	19	30	23	15
AT	12	16	27	21	14
C	1	0	1	0	0
KS	3	3	2	2	1

*NRPS* Non-ribosomal peptide synthetases, *PKS* Polyketide synthases, *DMATS* Dimethylallyltryptophan synthase, *AT* Acyl transferase, *C* Condensation, *KS* Beta-ketoacyl synthase.

While the PKS gene sequences identified by SMIPS could be useful to figure out which secondary metabolites each fungus might be able to produce, if there is not a close relative genome available with well-annotated gene clusters for production of a specific natural product, it is very difficult to determine which product is produced. Unfortunately, there are no tools that reliably predict the natural product from the gene sequences. We bumped into this impediment as four of the Ascomycota genomes (*Coniochaeta, Embellisia, Chaetomium* and *Phoma*) did not have close near neighbor genomes to which to compare. In spite of this, we identified some likely secondary metabolites that each fungus might produce, based on other members of their genus, and descriptions of the known secondary metabolites and toxins produced by related fungal endophytes and plant pathogens, where the biosynthetic gene clusters are known [47, 50, 54–61](Additional file 18). *Aspergillus* secondary metabolite query sequences were from the *A. fumigatus* Af293 genome (NC_007201.1), and the previously reported biosynthetic gene clusters from *A. fumigatus* [47, 49, 55]. The Aspergillus CK392 genome had high identity hits (generally > 90%) to all of the *A. fumigatus* Af293 query sequences, except fmtI (AFUA_8G00260) in the Fumitremorgin B cluster, where the hit had 67% identity to the query sequence, and the conserved hypothetical protein in the endocrocin gene cluster (AFUA_4G00225, 34% identity). The hits to all of the *A. fumigatus* Af293 query sequences are listed in Additional file 18 ‘Aspergillus SMs’ tab. The high % identity hits matching each *A. fumigatus* gene cluster (for the secondary metabolites endocrocin, fumagillin, fumiquinazoline; fumigaclavine C, fumitremorgin B, gliotoxin, hexadehydroastechrome, neosartoricin, fumicycline A, pesl, pes3 and siderophore) were sequentially located in the Aspergillus CK392 genome.

As two of the Ascomycota isolates in this study were provisionally determined to be related to *Phoma* and *Chaetomium* via ITS analysis, we used queries for secondary metabolite biosynthetic genes in *Phoma* and *Chaetomium* genomes to see if the FGC_4 (putative *Chaetomium* CK152) and FGC_5 (putative *Phoma* CK108) genomes had any similar biosynthetic gene sets. The queries included the biosynthetic gene clusters that produce diterpene aphidicolin in *Phoma betae*, squalestatin S1 in *Phoma* sp. MF5453 and chaetocin in *Chaetomium virescens* (Additional file 18 ‘Phoma, Chaetomium SMs’ tab). However, none of the genomes in our study had any high identity hits to these sequences, so it is unlikely that they can produce the natural products.

As all five of the fungal isolates appeared dark in culture, we examined their genomes for specific gene sets involved in melanin biosynthesis; melanin is an important pigment in fungi adapted to arid conditions [9], and is also associated with virulence [62]. Table 4 lists the genes present in each genome that had > ca. 50% identity with genes involved in the biosynthesis of three types of melanin that are commonly found in fungal cell walls: 1) DHN melanin, which is synthesized by gene clusters that include PKS enzymes [63–65]; 2) eumelanin, which is synthesized via L-DOPA by tyrosinase and tyrosinase-like proteins [66]; and 3) pyomelanin, which can be made from the L-tyrosine degradation pathway by some fungi [67]. From the results in Table 4, it appears that all five fungi have the genetic capability to make at least two of the three types of melanin. However, the actual ability of each fungus to make each type of melanin will need to be confirmed in culture studies [64, 65].

**Table 4:** Melanin Biosynthesis Genes

Genome	FGC_1 *Aspergillus* CK392	FGC_2 *Coniochaeta* CK134	FGC_3 *Embellisia* CK46	FGC_4 *Chaetomium* CK152	FGC_5 *Phoma* CK108
PKS gene cluster Query sequences	DHN melanin biosynthesis (*A. fumigatus* Af293)	DHN melanin biosynthesis (*Botrytis cinerea*)			
alb1(PKS)	g8340.t1 (99%)	g5144.t1 (60% id with Bcpks12, 46% id with Bcpks13)	g6238.t1 (49% id with Bcpks12 and Bcpks13)	g7880.t1 (54% id with Bpks12)	g4227.t1 (49% id with Bpks12 and Bpks13, 45% id with alb1)
arp1(scytalone reductase)	g8339.t1 (100%)	g6728.t1 (51% id with Bcscd1 and Bcscd2, 58% id with arp1)	g718.t1 (51% id with arp1, 48% id with Bscd1)	g9110.t1 (54% id with Bcscd1, 58% id with arp1)	g4314.t1 (48% id with Bcscd1, 53% id with arp1)
arp2 (hydroxynaphthalene reductase)	g8338.t1 (100%) g3356.t1 (55% id with *B. cinerea* Bcbrn1)	g9077.t1 (59% id with Bcbrn1 Condensin complex subunit 2) g2251.t1 (78% id with Bcbrn1) g5146.t1 (61% id with Bcbrn2 SDR)	g2727.t1 (52% id with arp2) g2550.t1 (54% id with Bcbrn1) g6236.t1 (69% id with Bcbrn1) g2727.t1 (59% id with Bcbrn2)	g10828.t1 (59% id with Bcbrn1) g11454.t1 (79% id with Bcbrn1) g11454.t1 (50% id with arp2) g5031.t1 (58% id with Bcbrn2)	g2131.t1 (53% id with arp2) g1699.t1 (52% id with Bcbrn1) g4229.t1 (70% id with Bcbrn1) g2131.t1 (60% id with Bcbrn2)
ayg1 (conidial pigment biosynthesis protein yellowish-green1)	g8337.t1 (99%)	Bcygh1 (Abhydrolase) g5555.t1 (64%)	g10909.t1 (50% id with *A. fumigatus* ayg1) g10909.t1 (54% id with Bcygh1)	g4919.t1 (60% id with Bcygh1) g4919.t1 (50% id with ayg1)	g8790.t1 (49% id with Bcygh1) g8790.t1 (51% id with ayg1)
abr1 (conidial pigment biosynthesis oxidase Abr1/brown 1)	g8336.t1 fragment (100% id last half of query) g8335.t1 (99%)	< 50% id	< 50% id	< 50% id	< 50% id
abr2 (conidial pigment biosynthesis oxidase/laccase)	g8334.t1 (99%)	< 50% id	< 50% id	< 50% id	< 50% id
Genome	FGC_1 *Aspergillus* CK392	FGC_2 *Coniochaeta* CK134	FGC_3 *Embellisia* CK46	FGC_4 *Chaetomium* CK152	FGC_5 *Phoma* CK108
Gene	Eumelanin biosynthesis				
Tyrosinase 1.14.18.1	g1264.t1	g1168.t1 g4438.t1	g6854.t1	g157.t1 g9197.t1	g5603.t1 g9014.t1 g9677.t1 g9249.t1 g145.t1 g784.t1 g2521.t1 g4858.t1 g2380.t1 g1694.t1
Genome	FGC_1 *Aspergillus* CK392	FGC_2 *Coniochaeta* CK134	FGC_3 *Embellisia* CK46	FGC_4 *Chaetomium* CK152	FGC_5 *Phoma* CK108
Gene	Pyomelanin biosynthesis				
Tyrosine aminotransferase tat/ aromatic aminotransferase 2.6.1.5/2.6.1.57	g5120.t1 g776.t1 (100% id, *Aspergillus fumigatus* Af293)	g2830.t1 g4574.t1 (84% id, *Coniochaeta ligniaria* NRRL 30616)	g10171.t1 g2384.t1 (92% id, *Alternaria alternata*)	g10445.t1 (95% id, *Colletotrichum salici*	g8502.t1 (90% id, *Epicoccum nigrum*)
4-hydroxyphenylpyruvate dioxygenase hppD 1.13.11.27	g986.t1 g4873.t1 g885.t1 g8201.t1 g6853.t1 g2062.t1 (*Aspergillus fumigatus* Af293)	g3403.t1 (80% id, *Coniochaeta ligniaria* NRRL 30616) g4879.t1 (92% id, *Coniochaeta ligniaria* NRRL 30616)	g5965.t1 (84% id, *Alternaria alternata*)g9210.t1(96% id, *Alternaria alternata*)	g935.t1 (80% id, *Coniochaeta ligniaria* NRRL 30616 g3716.t1 (87% id, *Thermothelomyces thermophila* ATCC 42464)	g2423.t1 (73% id, *Alternaria alternata*) g7746.t1 (93% id, *Ascochyta rabiei*)
auto-oxidation (spontaneous *oxidation* in air) step followed by polymerization into pyomelanin	+	+	+	+	+

+ Assume that this step can occur, since no enzyme is needed

Proteins relevant to environmental adaptation and competition include those involved in the production of mycotoxins. The presence of gene clusters for mycotoxin biosynthesis could be useful to distinguish saprotrophic fungi from plant pathogens. For example, *Coniochaeta* CK134 showed an increase in expression of aflatoxin B1-aldehyde reductase (Coniochaeta_CK134_g837.t1) under all growth conditions (grass, pine and chitin) (Additional file 12 ‘common pathways’ tab, Additional file 13). This enzyme may metabolize aflatoxin itself, or other charged aliphatic and aromatic aldehydes, which are toxic to cells [68]. Aflatoxin is a secondary metabolite, which can be pathogenic to humans, animals and plants [44, 69]. *Aspergillus* species are known to produce aflatoxin, and the aflatoxin biosynthesis gene clusters have been identified [47, 70, 71]. We used BLASTP [72] to search each genome for genes involved in aflatoxin biosynthesis. Additional file 18 lists the top candidate(s) in each genome that showed some sequence similarity to the aflatoxin biosynthesis gene cluster from *Aspergillus flavus* BN008 (GenBank accession number AY510452.1). Although many of the hits in the Ascomycota genomes had less than 50% identity to the query sequences, most of the hits were to proteins with similar annotated functions to the query sequences. However, aflatoxin biosynthetic capability cannot be inferred from these results. Experiments that demonstrate aflatoxin production will be necessary to confirm or refute this potential capability.

### Proteins involved in plant interactions

Fungi that interact with plants, either as beneficial partners, or as detrimental pathogens, produce proteins that aid them in these interactions. Fungal toxins, both host specific and non-host specific, have been described in numerous studies of plant pathogens [47, 50, 54, 56–61]. We compiled a list of the components involved in biosynthesis of these toxins, then acquired their sequences from fungal pathogen genomes, and used them as queries in blast searches of the five Ascomycota genomes (Additional file 18 ‘Toxins’ tab). In addition, some toxins were identified by examining the protein annotations for each genome (Additional file 19). From this comparison, Aspergillus CK392, Embellisia CK46 and Phoma CK108 had the highest numbers of potential toxin genes, at 35, 40 and 30, respectively (Additional file 18 ‘Toxins’ tab). Coniochaeta and Chaetomium had about half as many (15 and 14 genes, respectively). One interesting finding was that Chaetomium had no identifiable genes encoding allergen proteins, in particular alt a1, but the other genomes did. Allergen alt a1 and its homologs are characteristic of the Dothideomycetes and Sordariomycetes classes of fungi [73]. Alt a1 homologs can bind to plant plasma membranes and are perceived by the plant immune system [74]. Alt a1 is expressed during *Alternaria*-mediated plant pathogenesis, suggesting a possible virulence function [75], which might be to facilitate fungal pathogen interactions with plants. The *Aspergillus* CK392 genome encoded thirteen Alt a1-like allergens, as well as numerous proteins with homology to toxin biosynthesis components from phytopathogenic fungi [47, 50, 54–61].

The *Coniochaeta* CK134 genome did not encode any complete toxin biosynthesis gene clusters (Additional file 18). However, as the query sequences were from unrelated fungal genomes, this is not a definitive result. The *Coniochaeta* genome did encode two LysM domain proteins; one of these was expressed in all three culture conditions (g1171.t1), while the other was expressed in grass and pine cultures (g6810.t1). LysM domain proteins bind chitin, and are thought to participate in modification of cell walls by fungal plant pathogens to prevent plant recognition (reviewed by [57, 76]). *Coniochaeta* had one alt a1-like allergen (g4449.t1), which was expressed under all three conditions. The *Coniochaeta* CK134 genome also encoded candidate enzymes for oxalic acid metabolism (g5580.t1, g4635.t1, g7701.t1, g2391.t1), which could be involved in plant biomass degradation and has also been implicated in pathogenesis [77].

We did not identify any complete toxin biosynthesis gene clusters in the *Embellisia* genome. The *Embellisia* genome encoded two allergens; one of the allergens was expressed on all growth substrates (Embellisia_CK46_g9301.t1), and the other was expressed when the fungus was grown on chitin and pine (Embellisia_CK46_g9020.t1). Like *Embellisia* CK46, the *Phoma* CK108 genome encoded an elicitin (Phoma_CK108_g9101.t1), which was expressed during growth on all of the substrates (Additional file 18). Elicitins are secreted by fungal phytopathogens, provoking defense responses from plants [78]. The elicitins in *Embellisia* and *Phoma* were expressed during growth on all of the substrates (Additional files 2 and 3). *Embellisia*, *Chaetomium* and *Phoma* all had genes encoding cutinases, which are extracellular fungal enzymes that degrade cutin, which is a component of the waxy coating on plant leaves and shoots [79]; cutinases may be essential for the pathogenicity of certain fungal species to plants [80]. All three fungi expressed at least one cutinase under all conditions. *Embellisia* had four cutinases, three were expressed under all three conditions and one was not expressed under any condition (Additional file 3). *Chaetomium* had four cutinases, one was expressed when the fungus was grown in chitin and grass, two were not expressed under any condition, and one was expressed under all three conditions (Additional file 3). *Phoma* had two cutinases, one was expressed under all three conditions, and the other was not expressed under any condition (Additional file 3). The *Phoma* genome encoded two copies of allergen alt a1 (Phoma_CK108_g4015.t1, Phoma_CK108_g7594.t1), which were expressed under all growth conditions.

None of the genomes encoded any avirulence protein (Avr), a type of extracellular effector [81]. The *Aspergillus* and *Embellisia* genomes encoded proteins with homology to necrosis and ethylene-inducing peptides, which cause necrosis of plant tissues [82]. The one necrosis and ethylene-inducing-like protein in *Aspergillus* did not show an increase in expression above the sucrose control under any condition (Additional file 3). *Embellisia* had two of these proteins, one was expressed above the sucrose control under all three conditions, and the other showed increased expression when the fungus was grown in chitin and grass. Elicitins and necrosis and ethylene-inducing peptides can trigger plant defense responses, so our results suggest that *Aspergillus*, *Embellisia* and *Phoma* interact with plants.

In addition to toxins, small secreted proteins (SSPs) are produced by fungi that interact with plants [83]. SSPs are also called effector proteins, because they may participate in plant infections or in modulating plant responses to infection [83, 84]. SSPs have also been suggested to be involved in the degradative capabilities of saprophytic fungi and in other plant and microbial interactions [85]. Candidate small secreted proteins were identified in the fungal genomes using similar methods to those described by Ohm and colleagues [54]. Table 5 lists the number of proteins that were identified in each genome, using the criteria of having less than 200 amino acids, with a signal peptide identified by signalp (version 4.1) and no transmembrane helices (TMHMM version 2.0) [54].

**Table 5:** Candidate small secreted proteins in fungal genomes

Genome	FGC_1 *Aspergillus* CK392	FGC_2 *Coniochaeta* CK134	FGC_3 *Embellisia* CK46	FGC_4 Chaetomium CK152	FGC_5 Phoma CK108
Total number of SSPs^a^	132	187	205	274	167

^a^Candidate SSPs have less than 200 amino acids, have a signal peptide identified by signalp (version 4.1) and no transmembrane helices identified by TMHMM version 2.0 [54]

Only two candidate SSPs had homologs in all five fungal genomes, and both showed increased expression under some of the culture conditions (Additional file 12); these were allergenic cerato-platanin Asp F13 (Aspergillus_CK392_g2965.t1), which showed increased expression in all conditions in all fungi except *Aspergillus*, and 60S acidic ribosomal protein P2/allergen Asp F8 (Aspergillus_CK392_g6092.t1), which showed increased expression under some conditions in all fungi except *Aspergillus*). There was a third candidate SSP with homologs in all five genomes, that had a 201 amino acid protein sequence length (just above the 200 amino acid cutoff). This protein was annotated as isochorismatase family hydrolase (Aspergillus_CK392_g906.t1), and it showed increased expression in *Embellisia*, *Chaetomium* and *Phoma* under all three conditions. The numbers of SSPs in each fungal genome are listed in Table 5, and their annotations are presented in Additional file 16. Table 5 also shows the number of SSPs that were unique to each genome, and those that showed an increase in expression under any or all of the culture conditions. In *Aspergillus*, the nine SSPs that showed a change in expression included six hypothetical proteins, one 18 kDa antigen, one phosphoglycerate mutase family protein, and one secreted antimicrobial peptide (Additional file 16). The eight SSPs in *Coniochaeta* that showed a change in expression included six hypothetical proteins, one DNase1 protein and one PR-1-like protein. *Embellisia* had nine hypothetical candidate SSP proteins with a fold change and *Phoma* had two hypothetical candidate SSP proteins with a fold change. *Chaetomium* had the most (twenty) candidate SSPs showing a change in expression under any condition, including nineteen hypothetical and one glycoside hydrolase family 18 protein.

### CAZyme analysis

The CAZyme repertoire provides better clues about fungal lifestyles than the pathway analyses in Fig. 3, and Additional files 13 and 15. Proteins that contain CAZyme catalytic domains include glycoside hydrolases (GHs), polysaccharide lyases (PLs), carbohydrate esterases (CEs), and carbohydrate binding modules (CBMs), which indicate the ability to degrade particular plant cell wall polysaccharides. This approach has been used by others to separate fungal CAZymes into functional groups for degradation of the different types of plant biomass [86]. We used hmmsearch [87] to identify CAZymes in each genome by comparison of the protein coding sequences against the dbCAN database [88]. A summary of the total numbers of fungal proteins in each CAZyme category is shown in Additional file 20. Raw DbCAN and Pfam hits for each fungal genome are listed in Additional files 21 and 22. The *Embellisia* CK36 genome generally had higher numbers of proteins in each category, except for the PL category, where *Phoma* CK108 had more. The proteins that had hits to the CAZyme categories (Additional file 20) were grouped by plant substrate using categories that have been presented by others [86, 89, 90]. These results are summarized in Additional file 23. Each of the fungal genomes had multiple copies of genes encoding the enzymes for degradation of the plant materials cellulose, xyloglucan, xylan, galactomannan, pectin, starch, lignin and hemicellulose, and many of these proteins showed increased expression under one or more of the treatment conditions. Looking at Additional file 23 with respect to plant polysaccharide substrates, the highest numbers of these genes in all five genomes were in the categories of cellulose, hemicellulose and pectin degradation.

The fungal genomes encoded total numbers of plant biomass degrading enzymes that were within about 20% of each other (507, 584, 589, 644, 512). *Chaetomium* at 644 genes, had 21% more genes than *Aspergillus*’ 507 genes. Some general trends in protein expression can be seen from the CAZyme categories listed in Additional file 23. *Aspergillus*, *Coniochaeta* and *Embellisia* had similar total numbers of expressed plant polysaccharide degrading enzymes during growth on chitin, pine and grass. *Chaetomium* showed slightly lower total numbers of expressed proteins on pine (113 proteins), followed by chitin (136 proteins) and grass (153 proteins). *Phoma* expressed twice as many plant polysaccharide degrading enzymes on grass and pine (32 and 30 proteins, respectively) compared to chitin (14 proteins). In all of the CAZyme categories and under all three culture conditions, *Phoma* expressed the fewest proteins overall compared to the other fungi, while *Chaetomium* showed the highest expression. Each of the five fungal genomes encoded most of the enzymes involved in plant biomass degradation (Genome columns in Additional file 23). However, the expression of specific classes of these enzymes differed under the three culture conditions (chitin, grass, pine), and also varied across the isolates.

Cellulose is made up of hundreds to thousands of β-1,4 linked glucose units, with the disaccharide cellobiose as the repeating unit. Complete depolymerization of cellulose produces glucose [91]. Cellulose degradation involves the synergistic action of three classes of hydrolytic enzymes: 1) Endo-1,4-β-glucanases, which randomly cleave internal bonds in the cellulose chain, 2) Exo-1,4-β-glucanases (cellobiohydrolases), which attack the reducing or nonreducing end of the cellulose polymer, and 3) β-glucosidases, which convert cellobiose, the major product of the endo- and exo-glucanase mixture, to glucose [91, 92]. A recent discovery is that some fungal proteins with homology to CAZy family GH61 (multicopper oxidase, lytic polysaccharide mono-oxygenase, LPMO) exhibit cellulolytic enhancing ability when combined with common cellulases [91, 92].

Each of the Ascomycota genomes encoded the classic cellulose degrading enzymes, as well as many LPMOs. At least one endo-1,4-β-glucanase (either β-1,4-endoglucanase (GH5, GH7, GH12, GH45) or endoglucanase/xyloglucanase (GH9,GH44,GH45,GH74), or both, were expressed by all of the fungi except *Coniochaeta* under every culture condition. Exo-1,4-β-glucanases (cellobiohydrolases, GH6, GH7) were expressed in multiple copies by *Chaetomium* under all culture conditions. *Coniochaeta* and *Embellisia* each expressed one copy in chitin and grass cultures, while *Aspergillus* and *Phoma* did not express this enzyme at all. *Coniochaeta*, *Embellisia* and *Chaetomium* expressed at least one copy of β-glucosidase (GH1, GH3) on all substrates, while *Aspergillus* and *Phoma* each expressed at least one copy in grass and pine cultures. *Embellisia* and *Chaetomium* expressed multiple LPMOs (AA9, AA10, AA11, AA13**)** on all substrates and *Phoma* expressed one LPMO on all substrates. *Aspergillus* and *Coniochaeta* did not express any LPMOs under any condition.

Hemicelluloses are non-cellulose heteropolymers with varying degrees of branching. Different types of hemicelluloses are characteristic of different types of plants. Xylan is abundant in grasses and hardwood trees, mannan is found in softwoods like pine, and xyloglucans are abundant in many angiosperms. Galactomannans are another component of hemicellulose. Depending on the plant source and type of hemicellulose, degradation of hemicelluloses produces mixtures of different sugars [91, 93].

Fungi can use both nonspecific and specific types of endo-β-(1 → 4)-glucanases for hydrolyzing xyloglucan polymer backbones [91, 93]. These enzymes belong to the GH5, GH12, GH16, and GH74 CAZyme families. Xylan degrading families include GH10, GH11, and GH30. In the absence of GH30 xylanases, β-xylosidases in families GH3, GH43, and GH54 can substitute those functions. Mannanases can be in the GH5, GH7 GH8, and GH26 CAZyme families. GH26 also contains enzymes with β-1,3-xylanase activity. β-mannosidases, which hydrolyze β-1,4-mannosidic linkages in mannans, galactomannans and glucomannans [94] can belong to GH1 or GH2 families.

All of the fungal genomes encoded multiple xylanase genes, including a β-1,4-endoglucanase (GH5,GH7,GH12,GH45), which was expressed in all of the fungi except *Coniochaeta*, under all three conditions, a xyloglucan β-1,4-endoglucanase (GH12,GH74), which was expressed in all but *Coniochaeta* and *Phoma*, under all three conditions, an enzyme annotated as endoglucanase/xyloglucan hydrolase/β-1,3-1,4-glucanase/β-xylosidase (GH12), expressed in *Aspergillus* and *Embellisia* under all three culture conditions, but was not expressed by the other fungi. Multiple xyloglucanases of the GH16 family were expressed in all five fungi, under all conditions. An endoglucanase/xyloglucanase (GH9, GH44, GH45, GH74) was expressed in *Embellisia* and *Chaetomium* under all three conditions, and *Phoma* in grass. Multiple copies of β-1,4-endoxylanase (GH10, GH11) were expressed in *Embellisia* and *Chaetomium* under all three conditions, and one copy of this enzyme was expressed in *Coniochaeta* in grass cultures. Multiple GH30 family enzymes were expressed by *Coniochaeta* on all substrates, and one copy was expressed by *Chaetomium* on all substrates. Multiple β-1,4-glucosidases (GH1, GH3) were expressed by *Coniochaeta*, *Embellisia*, and *Chaetomium* on all of the substrates, while *Aspergillus* and *Phoma* expressed 1 and 2 copies, respectively, in grass and pine. At least one α-arabinofuranosidase (GH51, GH54) was expressed by *Aspergillus, Embellisia* and *Chaetomium* on all substrates. Multiple β-1,4-xylosidases (GH3, GH43) were expressed by *Aspergillus*, *Coniochaeta*, *Embellisia* and *Chaetomium* in chitin, grass and pine cultures. *Phoma* expressed two of these proteins in grass and pine cultures. β-xylosidase/α-L-arabinofuranosidase/arabinose/xylanase (GH43) was expressed by *Aspergillus*, *Coniochaeta*, and *Embellisia* in chitin, grass and pine cultures, and in *Chaetomium* pine cultures.

Each genome encoded multiple candidate mannanases in the CAZy families GH5, GH7 GH8, and GH26. Because some of the CAZyme families include multiple activities, some of these proteins were described above as candidate xylanases and cellobiohydrolases. The GH8 β-1,4-endomannanase was not encoded in any of the genomes, so was not expressed by any of the fungal isolates. All of the fungi except *Coniochaeta* expressed at least one copy of β-1,4-endomannanas (GH5, GH26) in chitin, grass and pine cultures. *Coniochaeta*, *Embellisia*, and *Chaetomium* did not express any GH1 family mannosidases, but *Aspergillus* expressed one of these enzymes, and *Phoma* expressed two in grass and pine cultures. *Aspergillus* expressed β-1,4-mannosidase (GH2) on all three substrates, *Chaetomium* expressed this enzyme on chitin and grass, and the other isolates did not express it.

Pectins can have very different structures, depending on the plant of origin, so the list of pectinolytic enzymes in Additional file 23 is from multiple sources [19, 86, 89, 90, 93, 94]. Since we don’t know exactly which type of pectin, if any, was present in each of the chitin, grass and pine substrates, a general comparison is presented here. Each of the fungal genomes encoded all of the pectinolytic enzymes listed in Additional file 23. *Aspergillus* expressed seven of them in all three culture conditions, *Coniochaeta* expressed four of the enzymes in all three culture conditions, and one in chitin cultures. *Embellisia* expressed eight of the pectin-degrading enzymes under all three conditions, one each in chitin and grass cultures, and four in pine. *Chaetomium* expressed nine of the enzymes under all three conditions, two in pine cultures and two in grass cultures. *Phoma* expressed only two pectin degrading enzymes under all three conditions, as well as two in grass and pine cultures.

Each of the genomes encoded four starch degrading enzymes. *Coniochaeta* and *Chaetomum* expressed all four of the starch degrading enzymes in chitin, grass and pine cultures. *Aspergillus*, *Embelisia* and *Phoma* each expressed three. These were glucoamylase (GH15), which was expressed under all three conditions, α-1,4-glucosidase (GH31), expressed under all three conditions in *Aspergillus*, and expressed in grass and pine cultures by *Phoma*, and inulinase (GH32) expressed in grass and pine by *Aspergilllus* and *Phoma*. *Embellisia* expressed α-amylase (GH13), α-1,4-glucosidase (GH31) and inulinase (GH32) in all three culture conditions.

Lignin degradation was an unpopular category among these fungi. The *Aspergillus* genome encoded all but one (Pyrroloquinoline quinone-dependent oxidoreductase, AA12) of the ligninolytic enzymes listed in Additional file 23. However, *Aspergillus* did not express any lignin degrading enzymes under any condition. The rest of the genomes encoded all of the lignin degrading enzymes but expressed very few of them. *Coniochaeta* only expressed one lignin degrading enzyme, laccase/multicopper oxidase (AA1), and only in chitin and pine cultures. *Embellisia* expressed four ligninolytic enzymes and *Chaetomium* expressed three, in all three culture conditions.

### Growth-related proteins

Most fungi grow through the extension of hyphae, which are fiber-like structures made of one or more cells encased within a single, long cell wall [95]. Components needed for fungal growth include vesicles containing biomolecules, that are continuously transported by cytoskeletal motor proteins to the hyphal tip [96]. The complex, dynamic, cross-linked fungal cell wall is comprised of chitin, glucans, other polysaccharides and proteins [97]. Chitin, an important polysaccharide component of fungal cell walls, is synthesized by members of a family of chitin synthases, which can be carried to growing hyphal tips by vesicles. A recent paper reports using super-resolution microscopy to observe secretory vesicles carrying the class III chitin synthase ChsB to the hyphal tip of *Aspergillus nidulans* [96]. ChsB plays a key role in hyphal tip growth, maintenance of cell wall integrity, and development [98]. There are seven classes of fungal chitin synthase enzymes, suggesting functional redundancy in cell wall-related functions; the expression and activity of chitin synthases is regulated during the cell cycle [99].

The dbCAN (Additional file 21) and Pfam (Additional file 22) hits included numerous proteins in all five fungi with chitin synthase and chitin binding domains. The Pfam domains with these functions are ‘Chitin_bind’ and ‘Chitin_synth’. The CAZy carbohydrate binding modules for chitin are CBM1, CBM2, CBM3, CBM5, CBM12, CBM14, CBM18, CBM19, CBM37, CBM50, CBM54, CBM55. All five fungal genomes had proteins with CBM1, CBM18, and CBM50 domains; and *Embellisia, Chaetomium* and *Phoma* had proteins with CBM19 and CBM37 domains.

There were numerous proteins with ‘Chitin_bind’ Pfam domains in the unique genes lists for all of the fungal genomes (Additional file 16), but only a few of these showed increased expression under any of the growth conditions: Phoma_CK108_g9791.t1 (all three conditions), Chaetomium_CK152_g1855.t1 (chitin and grass), Chaetomium_CK152_g2423.t1 (grass), and Embellisia_CK46_g5944.t1 (all three conditions). There was one chitin synthase G homolog present in all fungal genomes, that showed increased expression in *Chaetomium* grown in grass (Additional file 12).

Vesicular transport proteins with homologs in all of the fungal genomes that showed increased expression in some fungi under some conditions included: vesicle fusion ATPase, Arf, SNAP, synaptobrevin, VPS25/ESCRT-II, and VPS28. Arf (Coniochaeta_CK134_g8070.t1) and SNAP (Coniochaeta_CK134_g1809.t1) showed increased expression in *Coniochaeta* under all conditions, VPS25/ESCRT-II (Coniochaeta_CK134_g5217.t1) and VPS28 (Coniochaeta_CK134_g5098.t1) had increased expression in *Coniochaeta* in chitin and grass, respectively. *Embellisia* Arf (Embellisia_CK46_g3164.t1) increased in chitin, while in *Phoma* Arf (Phoma_CK108_g8441.t1) showed increased expression in grass.

*Chaetomium* vesicle fusion ATPase (Chaetomium_CK152_g6996.t1), Arf (Chaetomium_CK152_g10659.t1), SNAP (Chaetomium_CK152_g6631.t1), and synaptobrevin (Chaetomium_CK152_g8352.t1) showed increased expression during growth in chitin (Additional file 3). All of the fungal genomes had cytoskeleton proteins including actin, dynein, kinesin and tubulin, and some of them showed increased expression under one or more conditions (Additional files 12 and 16).

Phosphate solubilizing fungi in the soil can increase the bioavailability of soil phosphates for plants, and they do this by several mechanisms (reviewed by [100]). One mechanism is through the release of organic acids into the soil, which reduces the pH and can bring insoluble forms of phosphate into solution, where it is available for plants to accept. Other strategies for solubilizing organic phosphate involve the actions of phytases and phosphatases, which release phosphate from phytic acid and other phosphorus-containing compounds in the soil. While the main organic acids produced by industrially useful fungi are known [101], the ones produced by the fungi in these experiments are unknown, as they were not measured in the culture media. However, all of the Ascomycota genomes contained genes encoding organic acid biosynthetic enzymes, as well as phytases and various phosphatases, so it is possible that these fungi play roles in soil phosphate solubilization.

## Discussion

The Ascomycota fungi described here were isolated from different soil crust microhabitats (lichen, moss, and cyanobacteria-dominated biocrusts) and rhizosphere soils around the native bunchgrass *Hilaria jamesii* in an arid grassland near Moab, UT, USA (Ndinga Muniania et al. 2019, in review; Albright et al. 2019, in review) [1, 8, 9]. *Coniochaeta* CK134 was isolated from lichen biocrust, *Embellisia* CK46 from cyanobacteria biocrust*, Chaetomium* CK152 was from below lichen biocrust and *Phoma* CK108 was isolated from the moss microhabitat. *Aspergillus* CK392 came from the same soil environment as one of the most common fungi found during the isolation process. The fungi were grown in replicate cultures on different carbon sources (chitin, native bunchgrass or pine wood), which are relevant to carbon decomposition in soils, then the genomes and secretomes produced on each substrate were characterized.

Our results demonstrate that the five Ascomycota fungi from arid grassland soils are likely DSEs that secrete a wide range of proteins with potential roles in beneficial and detrimental interactions with plants and biocrust, including enzymes that degrade plant organic matter, small secreted effector proteins, and proteins that may be involved in virulence functions. We also identified proteins involved in fungal growth and metabolism, supporting previous results that DSF from arid soils show interspecific functional metabolic diversity [102].

### Dark septate endophytes

All five of the fungi appeared darkly pigmented in culture. While *Aspergillus* is not considered to be a DSF, *Aspergillus* condia can have melanin as one of the cell wall components [103]. The other four isolates are likely DSEs. Dark septate endophytes (DSEs) are frequent root colonizers in many environments [10]; they are especially common in environments with strong abiotic stress, such as arid ecosystems [1, 2]. DSEs perform a variety of functions that can be either beneficial or detrimental to plant health. DSEs are often observed in the root zones of plants in arid and semi-arid environments [9, 104–106]. Fungi can employ various schemes to interact with host plants through different mechanisms including mutualistic, saprotrophic, necrotrophic, biotrophic, and hemibiotrophic relations [57]. In association with plant roots, DSEs can help plants overcome stress [107, 108], and facilitate nutrient mobilization and uptake [109]. A meta-analysis of plant responses to DSEs showed that inoculation with DSE fungi significantly increased total plant biomass and shoot N and P contents [110]. DSEs can also produce antibacterial and antifungal secondary metabolites to protect plants from pathogens and herbivores [11, 111, 112], while other secondary metabolites may facilitate pathogenic interactions with plants [47, 50, 54–61]. As the fungi in this study were isolated from soil microenvironments, they are likely not human pathogens. However, in some cases they may act as opportunistic pathogens in mammals or plants [1, 10, 11, 110, 113–115].

In confirmation of their dark appearances in culture (hyphae or conidia), all five of the Ascomycota genomes contained candidate gene sets for the biosynthesis of three types of melanin. Melanins are secondary metabolites, black or dark brown in color, and their molecular structures are diverse [62, 116–119]. Fungi can produce a variety of melanins from phenolic precursors, including eumelanins (black or dark brown), pheomelanins (yellow or red), soluble piomelanins and those formed from dihydroxynaphthalene compounds (DHN) [120, 121]. The major melanin type synthesized by fungi is 1,8-dihydroxynaphthalene (DHN) melanin, which is synthesized from acetyl-coenzyme A via a polyketide biosynthetic pathway [64]. Some fungi can produce the black pigment eumelanin via a dihydroxyphenylalanine (DOPA) dependent pathway, in which tyrosinases or laccases hydroxylate tyrosine via DOPA to produce dopaquinone, which auto-oxidizes and polymerizes to form eumelanin. Fungi that can produce eumelanins include *Neurospora crassa*, *Podospora anserina*, *A. nidulans*, *A. oryzae* and the pathogen *Cryptococcus neoformans* [122]. Another type of fungal melanin, pyomelanin, is produced from L-tyrosine through 4-hydroxyphenylpyruvate and homogentisic acid [67, 120, 122]. *A. fumigatus*, *Madurella mycetomatis* and *Yarrowia lipolytica* are examples of fungi that can produce this type of pigment. As listed in Table 4, all five Ascomycota fungi had candidate gene sets to produce all three types of melanin. Melanin may protect these fungi against the harsh environmental conditions (reviewed by [120, 122]) in their arid environment. Fungal melanin may also play a role in plant pathogenesis [119]. Melanized fungal structures can penetrate plant tissues, allowing host invasion [119]. Examples of fungal plant pathogens that rely on this process to cause disease include *Colletotrichum kahawae,* which causes coffee berry disease [123], *Magnaporthe grisea,* the cause of rice blast [124] and *Diplocarpon rosae*, which causes black spot rose disease [125].

### Plant interactions

Our results provide evidence for fungal-plant interactions, mediated through SSPs, the fungal cell wall, plant biomass degrading enzymes, and other proteins that facilitate interactions with plants. We identified genes encoding numerous SSPs in all five fungal genomes using similar methods to [54, 85]. Many of the identified candidate SSPs had no sequence similarity to known proteins. SSPs may play roles in fungal-plant interactions [54, 57, 85, 126–129], although as small proteins their functions may not always be known [83]. SSPs may participate in manipulating plants as effectors, which likely play a role in host specialization and lifestyle [57, 83].

The fungal cell wall is an important structure, as it undergoes extensive remodeling and reorganization during fungal growth and hyphal extension [95, 130]. The fungal cell wall participates in beneficial plant interactions [131] and also interacts with plant tissues during infection [132]. Chitin is a core component of the fungal cell wall, performing structural functions during growth and infection-related changes. Chitin synthases, chitinases and other chitin binding proteins are important for these processes [99, 133, 134]. Our analyses of the five Ascomycota genomes identified multiple chitin-binding proteins and chitin synthase enzymes, along with components of vesicular transport, which facilitate growth of fungal hyphae and delivery of chitin synthases to the growing tips, where they add chitin to the cell wall [96, 133]. In addition to participating in cell wall morphogenesis during growth and infection, chitin-derived molecules may participate in signaling between mutualist species, whereby fungi secrete chitin-derived signaling molecules to prepare their hosts for the mutualistic relationship and the host plant responds to the signals [133].

Plant biomass, often termed lignocellulose, primarily consists of the energy rich structural polymers cellulose, hemicellulose and lignin, and also includes pectin, protein, low molecular weight compounds and ash [135, 136]. Cellulose is the most abundant polymer in softwoods, accounting for 45–50% of the biomass [135]. Grasses contain less cellulose (25–40%) than wood. Hemicelluloses are the second most abundant polymer, making up 35–50% of the biomass in grasses, and 25–35% in softwoods. Soft woods (such as pine) typically contain mannan hemicellulose, but grass has little of this type [137]. The lignin content of softwood ranges from 25 to 35%, and in grasses lignin comprises 10–30% of the biomass. Moreover, the chemical bonds in grass lignin are the same as those in wood lignin [137]. Biomass degrading microbes (bacteria and fungi) produce and secrete combinations of enzymes that act together to break down lignocellulose in plant cell walls [136].

To categorize potential genes encoding plant biomass degrading enzymes, we identified CAZymes in each genome by comparison of the protein coding sequences against the dbCAN database [88]. The total numbers of CAZymes involved in plant biomass degradation, that were predicted in the five fungal genomes, ranged from 507 in *Aspergillus* to 644 in *Chaetomium*. Overall, *Aspergillus* and *Phoma* expressed more CAZymes when grown in grass- and pine-containing cultures than in chitin. While *Aspergillus* expressed only three more CAZymes when grown in the presence of grass and pine, *Phoma* expressed twice as many. *Coniochaeta* expressed similar numbers of CAZymes under all conditions, (49 in chitin, 46 in grass and 48 in pine cultures). *Embellisia* expressed 87 CAZymes when grown in chitin and pine cultures, and 83 in grass. *Chaetomium* seemed to greatly prefer growing in cultures containing grass, where it expressed 153 CAZymes, compared to 136 in chitin and 113 in pine cultures.

Additional file 20 summarizes the numbers of CAZymes that were identified in each of the fungal genomes and Additional file 21 lists the accompanying dbCAN hits for more detail. Many of the CAZyme classes represent functions that participate in degradation of the plant biomass components lignin, cellulose, hemicelluloses, pectin and starch (Additional file 23). With respect to lignin degradation, all five Ascomycota species had multiple copies of proteins containing AA1 (laccases), AA2 (lignin peroxidases), AA3 (cellobiose dehydrogenase and various oxidases), AA4 (vanillyl-alcohol oxidase), AA5 (copper radical oxidases), AA6 (1,4-benzoquinone reductases) and AA8 (iron reductase) domains, which are CAZyme classes involved in lignin degradation [86], so all of the fungi likely have the genetic capability to degrade lignin-containing plant materials. None of these proteins showed an increase in expression in *Aspergillus* under any condition. The rest of the fungi showed increases in expression of some of these proteins under some of the conditions.

Each of the fungal genomes had multiple copies of genes encoding the enzymes for degradation of the plant materials cellulose, hemicelluloses (xyloglucan, xylan, galactomannan, mannan), pectin, starch, and lignin. All five genomes had multiple genes with CAZyme domains linked to cellulose degradation, but only *Embellisia* and *Chaetomium* expressed all of the cellulose degrading enzymes. *Coniochaeta* did not express any endoglucanase, while *Aspergillus* and *Phoma* did not express any exoglucanase (cellobiohydrolase). All of the isolates expressed β-1,4-glucosidase; *Coniochaeta*, *Embellisia* and *Chaetomium* expressed at least one β-glucosidase protein (GH1, GH3) on all substrates; while *Aspergillus* and *Phoma* each expressed at least one copy in grass and pine cultures. *Embellisia* and *Chaetomium* expressed multiple LPMOs (AA9, AA10, AA11, AA13**)** on all substrates, and *Phoma* expressed one LPMO on all substrates. *Aspergillus* and *Coniochaeta* did not express any LPMOs under any condition.

All of the fungal genomes encoded all of the enzymes necessary for degradation of the different types of hemicellulose: xylan, xyloglucan, mannan, and galactomannan. Enzymes for xylan, xyloglucan and mannan/galactomannan degradation were expressed in all of the fungi under all of the culture conditions. *Chaetomium* and *Embellisia* expressed the most pectinolytic enzymes (twelve and eleven, respectively, under any condition), while *Phoma* only expressed three pectin degrading enzymes under any condition. All of the fungal genomes encoded enzymes for starch degradation, and each fungus expressed at least three of the four types in at least two of the culture conditions.

All of the genomes except *Aspergillus* encoded all of the enzymes for lignin degradation listed in Additional file 23, and *Aspergillus* was only missing one enzyme in this category (Pyrroloquinoline quinone-dependent oxidoreductase (AA12)). Somewhat surprisingly, *Aspergillus* did not express any of the lignin degrading enzymes under any condition. This result is surprising, because some *A. fumigatus* strains can degrade lignin from various plant sources in culture [138, 139]. The expression of lignin degrading enzymes was low in all of the fungi. Lignin degradation has long been associated mainly with Basidiomycota, in particular white rot fungi. Ascomycota are generally thought to be unable to degrade lignin, and their genomes often lack most of the traditional lignin related oxidases [140]. However, some Ascomycetes can grow on lignin [138–140], and their genomes encode laccases and other lignin oxidative enzymes [140]. The numbers of lignin degrading proteins expressed in the presence of the different substrates was not very different. One explanation for this observation in the grass and pine cultures is that grass and pine lignin have a similar structure [137]. An initially puzzling result was the expression of lignin degrading enzymes in the chitin cultures of *Coniochaeta*, *Embellisia*, *Chaetomium* and *Phoma*. However, there is evidence that pure chitin is decomposed more rapidly than pure cellulose when added to soil, and it may be decomposed preferentially over other cell wall components [141]. Another explanation is that chitin and the cellulose from plant cell walls have structural similarities [142], so the enzymes that degrade the cellulose in grass and pine sawdust might also be able to promiscuously degrade chitin. CAZymes with more general functions (AA families) may aid other CAZymes in degrading complex substrates like lignin, which is frequently found in tight association with other polysaccharides in plant cell walls [143]. For example, LPMOs (CAZy families AA9,AA10,AA11,AA13), which were encoded by all five Ascomycota genomes, and expressed in cultures containing chitin, grass and pine substrates by *Embellisia*, *Chaetomium* and *Phoma*, can depolymerize various plant-derived substrates, like cellulose and hemicellulose [144, 145].

These results indicate that fungal pathways involved in plant biomass decomposition are activated during growth in the presence of chitin, grass and pine substrates. However, additional focused culture studies will be needed to determine the activities of the different enzymes in the presence of the various plant polysaccharide components, such as cellulose, hemicellulose, pectin, starch and lignin.

### Lifestyles of the DSEs

To determine the lifestyles of the arid soil DSEs, comparisons to related species is necessary. However, only the *Aspergillus* CK392 genome had close relative genomes to which to compare. Through genome comparison, we determined that the *Aspergillus* CK392 genome contained all of the secondary metabolite gene clusters in *A. fumigatus* genomes [47, 55], so it is likely a member of the *A. fumigatus* species. *A. fumigatus* is a soil dwelling saprophyte that obtains nutrition from dead and decaying organic matter such as soils and compost piles, where it participates in carbon and nitrogen cycling [146]; *A. fumigatus* can also be pathogenic to plants, humans and animals. The *Aspergillu*s CK392 genome encoded over 500 enzymes involved in plant polysaccharide degradation (Additional file 23), and many of these were expressed in chitin, grass and pine cultures, indicating that this *Aspergillus* likely obtains its nutrition from plant biomass. The *Aspergillus* CK392 genome encoded thirteen allergens, as well as proteins with homology to toxin biosynthesis components from phytopathogenic fungi [47, 50, 54–61], so it may be able to obtain nutrition as a saprotroph, or it could be an opportunistic pathogen.

The other genera had no very close relative genomes to which to compare them, so identification of species-specific gene sets, including those that produce secondary metabolites will have to wait until more closely related genomes are sequenced. However, comparing our results with other published studies provided clues about their lifestyles and ecological roles in their arid habitat. *Coniochaeta species are often found in association with plants* [147, 148], *and they can* degrade lignocellulose in a variety of woody substrates [149, 150], corn stover [151], wheat straw, switchgrass [152], sawdust and coffee residues [153]. *Coniochaeta lignaria* can utilize many of the phytotoxic compounds present in treated grass substrates to enhance lettuce seed germination [154]. Because they are often found in association with plants, it is not surprising that some species of the genus *Coniochaeta* (anamorph: *Lecythophora*) can be pathogens of woody hosts, such as *Prunus* trees [155] and peach trees [156]. *Coniochaeta* species have been identified on coniferous host trees (148), and *Lecythophora (Coniochaeta) hoffmannii* is a soil- and plant-associated isolate that can be a facultative tree pathogen that causes soft rot [157]. However, *Coniochaeta* spp. are reported to be of low virulence on most hosts, and they often colonize dead tissue or invade previously infected, wounded, or senescent plant tissues [155, 158].

The *Coniochaeta* CK134 genome encoded all of the enzymes necessary for plant biomass degradation listed in Additional file 23. However, not all of these enzymes were expressed during growth in the presence of chitin, grass and pine substrates. The *Coniochaeta* CK134 genome did not encode any complete toxin biosynthesis gene clusters. However, as the query sequences were from unrelated fungal genomes, this is not a definitive result. The *Coniochaeta* genome did encode two LysM domain proteins; one of these was expressed in all three culture conditions (g1171.t1), while the other was expressed in grass and pine cultures (g6810.t1). LysM domain proteins bind chitin, and are thought to participate in modification of cell walls by fungal plant pathogens to prevent plant recognition (reviewed by [57, 76]). *Coniochaeta* had one alt a1-like allergen (g4449.t1), which was expressed under all three conditions. Alt a1 is expressed during *Alternaria*-mediated plant pathogenesis, suggesting a possible virulence function [75]. The *Coniochaeta* CK134 genome also encoded candidate enzymes for oxalic acid metabolism (g5580.t1, g4635.t1, g7701.t1, g2391.t1), which could be involved in plant biomass degradation and has also been implicated in pathogenesis [77]. From all of this evidence, we can conclude that *Coniochaeta* CK134 is likely involved in plant interactions, but whether it functions as an endophyte, a saprobe or an opportunistic pathogen in some circumstances will require further studies.

*Embellisia* spp. are known root colonizing DSEs [102] in a variety of ecosystems, including arid and semiarid ecosystems, which have strong abiotic stressors [10, 11, 129]. *Embellisia* currently has an unresolved taxonomy [15], but it is related to *Alternaria*, and is a member of the *Alternaria* complex, which includes saprobic, endophytic and pathogenic species [13]. *Embellisia* spp. endophytes can be isolated from various types of locoweed, where they promote locoweed growth and therefore aid swainsonine production [159, 160]. *Embellisia* endophytes are also associated with wheat progenitors grown in desert soil [161]. In addition, an *Embellisia* sp. is pathogenic to the herbaceous perennial forage legume standing milk-vetch in China [162].

The *Embellisia* genome encoded all of the enzymes involved in cellulose, hemicellulose, galactomannan, pectin, starch and lignin degradation, and some of these proteins were expressed in the different culture conditions. *Embellisia* expressed similar numbers of CAZymes in the chitin, grass and pine cultures. In the potential toxin category, the *Embellisia* genome encoded one elicitin, two allergens, four cutinases, and numerous proteins with homology to toxin biosynthesis components from phytopathogenic fungi [47, 50, 54–61]. However, we did not identify any complete toxin biosynthesis gene clusters in the *Embellisia* genome. One allergen was expressed by *Embellisia* on all growth substrates (Embellisia_CK46_g9301.t1), and the other was expressed when the fungus was grown on chitin and pine (Embellisia_CK46_g9020.t1). The elicitin (Embellisia_CK46_g1791.t1) was expressed during growth on all three substrates. Three of the four *Embellisia* cutinases (g11015.t1, g11159.t1, g4869.t1) were expressed under all three culture conditions and one (g11942.t1) was not expressed under any condition. These results indicate that *Embellisia* CK46 leads a life that includes degradation of plant derived substrates. It also likely participates in interactions with plants, and the evidence for potential pathogenicity is stronger in *Embellisia* than *Coniochaeta*. However, further evidence is needed to definitively determine *Embellisia’s* functions in the arid grassland environment.

*Chaetomium* endophytes are commonly found in the soil, air and on leaves and wood [163, 164]. *Chaetomium* spp. are also common in desert soils [165]. Cultured *Chaetomium* isolates from different origins show similar patterns of biomass production on plant cell-wall related polysaccharides [163]. Some *Chaetomium* spp. may be able to function as endophytes in the rhizosphere, opportunistically colonizing plant roots, but becoming weakly pathogenic when resources are limited and competition with other microbes is high [166]. As the *Chaetomium* genome encoded the most CAZymes, and the isolate expressed the most CAZymes in all three of the culture conditions, it likely makes its living degrading plant tissues, and might be an opportunistic pathogen under the right conditions.

*Phoma* spp. are root associated endophytes [167] that can occur in a variety of ecosystems [168], and associate with various types of plants, including pine, switchgrass, and rosette grass [164], wheat grown in desert soil [169], and cucumber roots [60]. *Phoma* and *Chaetomium* are part of seed microbiomes [170]. *Phoma* spp. can be pathogenic to monocots and dicots [171]. The *Phoma* CK108 genome encoded all of the enzymes involved in cellulose, hemicellulose, galactomannan, pectin, starch and lignin degradation, and some of these proteins were expressed in the different culture conditions, although *Phoma* expressed more CAZymes in the grass and pine cultures. It appears from these results that *Phoma* may prefer growing in grass and pine over chitin. Furthermore, *Phoma* expressed an elicitin, two allergens and a cutinase in all three conditions; these proteins may be associated with phytopathogenicity [74, 80, 172]. Like *Embellisia*, the evidence for pathogenicity is stronger in *Phoma* than in *Aspergillus*, *Coniochaeta* and *Chaetomium*. Whether any or all of the isolates function as endophytes, saprophytes or opportunistic pathogens will require further studies.

## Conclusions

Our analyses of the genomes and secretomes of the five Ascomycota isolates revealed melanized structures and the genetic capability to synthesize melanin, which is relevant to their survival in arid systems [1, 10, 104, 110, 114, 167]. All of the genera described in this report secreted numerous proteins, including functional categories involved in interactions with plants (CAZymes, proteases, lipases, and oxidoreductases, SSPs) [57, 83]. Because they all had broad capabilities for plant biomass degradation, some of the Ascomycota DSEs may be latent saprotrophs that colonize plants but become active in plant biomass degradation upon senescence or death of the host plant [173]. This could be a valid lifestyle for some or all of these fungi, as they all secreted extracellular enzymes with the ability to degrade lignocellulosic substrates, which would facilitate the penetration of plant cell walls for colonization [1]. The production of mycotoxins has also been associated with saprotrophic lifestyles, as mycotoxin natural products would inhibit other fungal competitors for plant-derived resources [1], or enable the fungi to attack plant cell walls in various ways [93]. However, due to the lack of close neighbor genomes to which to compare, we were unable to identify complete sets of mycotoxin biosynthesis genes in the non-*Aspergillus* isolates.

By characterizing the genomic features, metabolic potential and secretomes of arid grassland Ascomycota fungi, this study contributes important information to understand the different ecological roles that these fungi play. Our results support the conclusion that some or all of the isolates likely interact with plants. It is also likely that many or all of these fungi show high ecological plasticity, in that they may be able to serve multiple roles depending on the growth substrate or changing environmental conditions.

## Methods

### Culture

Five fungal species (*Aspergillus* CK392 (MK439477) *Chaetomium* CK152 (MH474117), *Coniochaeta* CK134 (MH473986), *Embellisia* CK46 (MH474310), and *Phoma* CK108 (MH473793) were previously isolated from biocrusts and rhizosphere soils in a semiarid grassland near Moab, Utah, USA as follows (Ndinga Muniania et al. 2019, in review). Rhizosphere soil samples were collected at about 5 cm depth from the exotic invasive *Bromus tectorum* and native bunchgrass *Hilaria jamesii*. Biocrust soil samples were obtained from biological soil crusts (biocrusts), which cover soil spaces between plants and included three main types: lichen-dominated biocrusts, cyanobacteria-dominated biocrusts, and moss-dominated biocrusts. For each biocrust type, quadrants of 10 × 10 cm were randomly selected in locations where the three types of biocrusts occurred adjacent to each other. Soil samples were obtained from the surface (1–2 cm depth) and 5 cm below surface using a paint scraper and avoiding the green upper part for moss samples. About 10g of soil was collected for each sample type and placed directly into individual plastic bags on ice before being shipped to Los Alamos National Laboratory. Fungi were isolated using a serial dilution technique in quadruplicate for every sample (672 plates). Soil dilutions of 10^− 2^ and 10^− 3^ were inoculated (1000 μL) on malt extract agar (MEA) plates (100 mm) with two antibiotics: streptomycin and tetracycline [50 μg/L] (MEA + A). Plates were incubated in the dark for three days at 25 °C and checked every day for growth. Inoculated plates (10^− 2^ dilution) from every microhabitat were scanned on both sides of the petri dish after two weeks of growth for image analysis. Colonies obtained in the plates (10^− 3^ dilutions) were transferred onto new MEA + A plates for isolation of pure colonies. Isolation efforts were focused on unique morphotypes and tissue from each pure isolate was taken for DNA extraction. Fungi were stored in sterile water for further experiments at the Western Illinois University Fungarium, Macomb, IL and at Los Alamos National Laboratory, Los Alamos, New Mexico USA (Ndinga Muniania et al. 2019, in review).

For this study, the fungi isolated from the different microhabitats were: *Chaetomium* CK152: below lichen biocrust; *Coniochaeta* CK134: lichen biocrust; *Embellisia* CK46: cyanobacteria biocrust; *Phoma* CK108: moss; *Aspergillus* CK392: generally from the soil. A couple of plugs from each stock fungal culture were added to 250 ml baffled flasks, each with 150 ml of basal medium [174]. Four replicate cultures were established for each of four different carbon sources: chitin (SIGMA Chemical Company, St. Louis, MO), ground up perennial bunchgrass (*Pleuraphis jamesii*), and pine wood sawdust, each at 1% w/v in 0.2% sucrose, as well as 0.2% sucrose as the control. Per liter, the basal media contained 2 g of NH4NO3, 2 g of KH2PO4, 0.5 g of MgSO4·7H2O, 0.1 g of CaCl2·2H2O, 1 mg of thiamine hydrochloride, and 10 ml of mineral solution. Mineral solution contained, per liter: 1.5 g of nitrilotriacetic acid, 3 g of MgSO4·7H2O, 0.5 g of MnSO4·H2O, 1 g of NaCl, 0.1 g of FeSO4·H2O, 0.1 g of CoSO4, 0.1 g of CaCl2, 0.1 g of ZnSO4·7H2O, 0.01 g of CuSO4, 0.01 g of AlK(SO4)2·12H2O, 0.01 g of H3BO3, and 0.01 g of NaMoO4·2H2O. Cultures were maintained for 14 days at room temperature.

After 14 days of culture, pellets and supernatants were harvested by centrifugation to separate fungal biomass from supernatant. Supernatant samples for all treatments were sent to EMSL for proteome analysis along with sucrose pellet replicates for each fungus. Replicate pellet samples were pooled to create a single composite pellet sample for each fungus. The MP Biomedicals FastDNA SPIN Kit for Soils was used to extract genomic DNA from the mycelia harvested from liquid broth cultures using the protocol provided by the vendor.

### Genome sequencing and annotation

Genomes were sequenced on a single lane of HiSeq2000 (Illumina, Inc., San Diego, CA), and assembled using Velvet version 1.2.10 [175] with 61 bp kmer length. Genomes were annotated using Augustus version 3.0.3 [176] as described in the Methods. The assemblies were not optimized. The assembled contigs for each of the fungal genomes are available as Additional files 24, 25, 26, 27 and 28. Gene prediction was accomplished using Augustus version 3.0.3 [176] with the nearest-neighbor gene model (of those included with Augustus) as a guide, as follows. FGC_1: *Aspergillus fumigatus*; FGC_2: *Chaetomium globosum*; FGC_3: *Fusarium graminearum*; FGC_4: *Chaetomium globosum*; FGC_5: *Chaetomium globosum.* The protein coding sequences for each of the genomes are provided in Additional files 29, 30, 31, 32 and 33.

Protein coding sequences were functionally annotated by BLASTP [72] against the preformatted nr database, and hmmscan (HMMER package version 3.1b2) [177] searches of the fungal protein coding sequences against the Pfam-A [178] and dbCAN [179] hmm databases. A match to the dbCAN database was counted if the hit had an *e*--value <= 1e--20 and the query protein sequence was > = 50 amino acids long. A function was automatically assigned to each fungal coding sequence based on the top BLASTP hit using an in-house script. These assignments are included in Additional file 19. To assign more specific functions, especially in genomes with no close near relatives, annotations were manually updated using the Pfam and dbCAN hits. This information is included in Additional files 3, 12, and 16, along with the protein expression data.

Orthologues common among all five fungal genomes were identified by clique analysis using the Species Paralogy and Orthology Clique Solver (SPOCS) program [180], which uses NCBI BLAST (73)to identify reciprocal best hits, and a maximum clique algorithm to identify the orthologs and paralogs. The data from this analysis are presented in Additional file 1. The SPOCS application is designed to identify an orthologous group of proteins as a clique made up of pair-wise reciprocal best hits. SPOCS returns the predicted orthologs and paralogs in a tab-delimited report, and optionally, in a self-contained HTML output with visualizations of the ortholog relationships [180].

### Sample preparation for mass spectrometry

#### Pre-digestion methods

Supernatant. Frozen supernatant samples were allowed to thaw and the protein was precipitated by adding 20% trichloroacetic acid (TCA) and incubated at − 20 °C overnight. The following day the samples were thawed and centrifuged at 4500 xg at 4 °C for 20 mins to pellet the protein. The supernatant was decanted and the protein pellet was washed 2 times with ice-cold acetone. The pellet was allowed to slightly dry and 100 μl of UPX Universal Protein Extraction buffer (expedeon, San Diego, CA) was added and water-bath sonicated into solution. Each sample was incubated at 95 °C for 5 mins to ensure reduction and solubilization of protein. The samples were then vortexed and sonicated for 2 mins, lightly spun to collect condensate and allowed to cool at 4 °C for 45 mins. The samples were then centrifuged at 15,000 xg for 10 mins.

Fungal Pellet. TissueLyser II system (Qiagen, Valencia, CA) trays were frozen at − 20 °C overnight. Two 3 mm stainless steel beads were added to each sample tube and placed in the TissueLyser, the frozen samples were ground for 2 mins at 30 Hz until powderized.

1 mL of UPX extraction buffer was added to each sample and a hand-held OMNI TH homogenizer (OMNI International, Kennesaw, GA) was used to homogenize the sample for 5 mins on ice. Aliquots (1 mL) of each homogenate were removed into fresh tubes and spun at 5000 xg for 10 min.

### Sample digestion

Filter Aided Sample Preparation (FASP) [181] kits were used for protein digestion (expedeon, San Diego, CA) according to the manufacturer’s instructions. Briefly, 400 μl of 8 M urea (all reagents included in the kit) was added to each 500 μl 30 K molecular weight cut off (MWCO) FASP spin column and up to 100 μl of the sample in UPX buffer was added, centrifuged at 14,000 xg for 30 mins to bring the sample all the way to the dead volume. The waste was removed from the bottom of the tube and another 400 μl of 8 M urea was added to the column and centrifuged again at 14,000 xg for 30 mins and repeated once more. 400 μl of 50 mM ammonium bicarbonate (provided) was added to each column and centrifuged for 20 mins, done twice. The column was placed into a new fresh, clean and labeled collection tube. Digestion solution was made by dissolving 4 μg trypsin in 75 μL 50 mM ammonium bicarbonate solution and added to the sample. Each sample was incubated for 3 h at 37 °C with 800 rpm shaking on a thermomixer with a thermotop (Eppendorf, Hamburg, Germany) to reduce condensation into the cap. The resultant peptides had 40 μl of ammonium bicarbonate solution added and then they were then centrifuged through the filter and into the collection tube at 14,000 xg for 15 mins. The filter then had another 40 μl of ammonium bicarbonate solution added and then they were then centrifuged through the filter again. The peptides were concentrated to ~ 30 μL using a SpeedVac. Final peptide concentrations were determined using a bicinchoninic acid (BCA) assay (Thermo Scientific, Waltham, MA USA). Each sample was diluted to 0.1 μg/μl and vialed for Mass Spectrometry analysis.

### Mass spectrometry

All data were collected on a LTQ Orbitrap Velos mass spectrometer (Thermo Electron, Waltham, MA) coupled to a Next-Gen 3 high performance liquid chromatography system (Agilent Corporation, Santa Clara, CA) through 75 um × 70 cm columns packed with Phenomenex Jupiter C-18 derivatized 3 um silica beads (Phenomenex, Torrance, CA). Samples were loaded onto columns with 0.05% formic acid in water and eluted with 0.05% formic acid in Acetonitrile over 99 min. Ten data-dependent MS/MS scans were recorded for each survey MS scan (70 K nominal resolution) using normalized collision energy of 35, isolation width of 2.0 m/z, and rolling exclusion window lasting 30 s before previously fragmented signals are eligible for re-analysis.

### MS/MS data search

The MS/MS spectra from all LC-MS/MS datasets were converted to ASCII text (.dta format) using DeconMSn [182], which attempts to assign the appropriate charge and parent mass values to an MS/MS spectrum. The data files were then interrogated via target-decoy approach [183], each organism against its specific genome file combined with typically observed contaminant proteins (Keratins, Trypsin, etc.) using MSGFPlus [184] using a +/− 20 ppm parent mass tolerance, partial tryptic enzyme settings, and a variable posttranslational modification of oxidized Methionine. All MS/MS search results for each dataset were collated into tab separated ASCII text files listing the best scoring identification for each spectrum.

### Data analysis

Collated search results were further combined into a single result file. These results were imported into a Microsoft SQL Server database. Results were filtered to below 1% FDR using an MSGF+ supplied Q-Value that assesses reversed sequence decoy identifications for a given MSGF score across each dataset. Filter passing results were reported in an Excel file. Using the protein references as a grouping term, unique peptides belonging to each protein were counted, as were all PSMs belonging to all peptides for that protein (i.e. a protein level observation count value). PSM observation counts reported for each sample that was analyzed. Cross-tabulation tables were created to enumerate protein level PSM observations for each sample, allowing low-precision quantitative comparisons to be made.

Spectral count data were averaged across the technical replicates for each fungus and each treatment; the means, standard deviations, standard errors were calculated in R. For each fungal dataset, the average of the replicates for each treatment condition were computed in Microsoft Excel. R was used to calculate the standard deviation, standard error, *p*-values and fold change in expression for each pairwise comparison among the treatment groups. For each fungus on each carbon substrate, the fold change of the average protein counts for each condition was calculated compared to each other condition and pairwise *p*-values were calculated (Additional file 3). Data were filtered to exclude proteins that showed fold change values of zero and p-values of zero (since –log_10_(0) is undefined). Proteins that were present in the sucrose pellet at > 25 counts were noted. R was used to to visualize the fold change results as volcano plots (Figs. 1 and 2). Volcano plots are a special type of scatterplot, useful for visualizing changes in protein (or gene) expression [185]. In Figs. 1 and 2, each protein is represented by a dot. To improve the visualization of expression changes, the axes are log2 (fold change of protein expression in chitin, grass or pine cultures compared to sucrose alone) vs significance of the fold change, which is represented on the plot as -log10 (*p* value). The log of the fold change is used so that changes in protein expression spread out from the center, and the -log10 (*p* value) ensures that the more significant values are toward the top of the plot. Therefore, the regions of interest are the points near the top of the plot that are at the far left- or far right-hand sides of the plot. These points show large magnitude fold changes (left and right) and high statistical significance (near the top). R was also used to create the bar plots in Additional file 13, and the heatmaps in Fig. 3 and Additional file 15.

### Pathway analysis

For each fungal genome, the annot8R program [186] was used to assign EC numbers to the protein sequences. KEGG gene identifiers and pathways were assigned to protein sequences using the EC number from the annot8r annotation by comparison to KEGG orthology data [187].

### Secondary metabolite gene cluster identification

The SMIPS program was used to identify secondary metabolite producing enzymes (‘anchor’ genes), which include polyketide synthases, non-ribosomal peptide synthetases and dimethylallyl tryptophan synthases [51].

### Identification of small secreted proteins (SSPs)

SSPs were identified in each genome by running SignalP [188] and TMHMM [189], and filtering the results to only include protein sequences that were less than 200 amino acids long, had a signal peptide as predicted by SignalP and no transmembrane domain identified by TMHMM.

## Supplementary information

**Additional file 1.** Protein homologs in all fungi. List of proteins encoded by genes that had homologs in all of the fungal genomes. This data was from the SPOCS clique analysis.

**Additional file 2.** Raw protein abundance data (from EMSL). Raw protein abundance data for each fungus, each condition, including all replicates.

**Additional file 3.** Statistics for volcano plots. Statistics and annotations for the proteins that showed a fold change in expression over the sucrose control under each growth condition.

**Additional file 4.** Labeled volcano plot chitin vs sucrose all fungi. Labeled volcano plot showing proteins with increased expression in all fungi in chitin cultures compared to sucrose control cultures.

**Additional file 5.** Labeled volcano plot grass vs sucrose all fungi. Labeled volcano plot showing proteins with increased expression in all fungi in grass cultures compared to sucrose control cultures.

**Additional file 6.** Labeled volcano plot pine vs sucrose all fungi. Labeled volcano plot showing proteins with increased expression in all fungi in pine cultures compared to sucrose control cultures.

**Additional file 7.** Labeled volcano plot *Aspergillus* CK392 all culture conditions. Labeled volcano plot showing proteins with increased expression in *Aspergillus* CK392 in chitin, grass and pine cultures compared to sucrose control.

**Additional file 8.** Labeled volcano plot *Coniochaeta* CK134 all culture conditions. Labeled volcano plot showing proteins with increased expression in *Coniochaeta* CK134 in chitin, grass and pine cultures compared to sucrose control.

**Additional file 9.** Labeled volcano plot *Embellisia* CK46 all culture conditions. Labeled volcano plot showing proteins with increased expression in *Embellisia* CK46 in chitin, grass and pine cultures compared to sucrose control.

**Additional file 10.** Labeled volcano plot *Chaetomium* CK152 all culture conditions. Labeled volcano plot showing proteins with increased expression in *Chaetomium* CK152 in chitin, grass and pine cultures compared to sucrose control.

**Additional file 11.** Labeled volcano plot *Phoma* CK108 all culture conditions. Labeled volcano plot showing proteins with increased expression in *Phoma* CK108 in chitin, grass and pine cultures compared to sucrose control.

**Additional file 12.** Protein expression among homologs in all fungi. Annotated proteins and their expression (fold change under each culture condition compared to sucrose control). The tab ‘common pathways’ includes proteins found in all five fungal genomes. ‘Selected pathways’ includes proteins with the most interesting changes in expression. ‘Not common pathways’ lists proteins with expression changes that were not present in all five fungal genomes.

**Additional file 13.** Fold change in expression of proteins, grouped by pathways. Barchart showing expression changes in each fungus, under each culture condition, grouped by metabolic pathways and other functional categories. The data for this Figure are provided in Additional file 12 ‘common pathways’ tab.

**Additional file 14.** Cliques report with protein abundances. Data summary that was obtained from Additional file 1, and Additional file 12 (‘selected pathways’ tab). These data were used to generate the heatmaps in Fig. 3 and Additional file 15.

**Additional file 15.** Heatmaps all replicates. Heatmap showing the expression levels of proteins with annotated functions in pathways for plant biomass degradation, defense and virulence (pathogenesis). Total protein counts in all replicates for each treatment condition are shown for each fungus.

**Additional file 16.** Unique genes list. For each fungal genome, a list of the protein coding genes that were not found in the other four fungi, their annotations and expression values.

**Additional file 17.** SMIPS output. Output of the SMIPS program (secondary metabolite anchor genes) for each fungal genome.

**Additional file 18.** Fungal toxins and secondary metabolites. Lists of fungal toxins ‘Toxins’ tab and secondary metabolites ‘Aspergillus SMs’ and ‘Phoma, Chaetomium SMs’ tabs, and evidence describing whether or not each of the fungal genomes encodes candidate genes for their biosynthesis.

**Additional file 19.** Annotated proteins. BLASTP top hits to the nr database for each of the coding sequences in each of the fungal genomes.

**Additional file 20.** CAZymes. Table listing the numbers of CAZyme hits in each CAZy family in each fungal genome.

**Additional file 21.** DbCAN hits. Table showing DbCAN hits in each fungal genome. The output of hmmscan.

**Additional file 22.** Table showing Pfam hits in each fungal genome. The output of hmmscan.

**Additional file 23.** CAZymes involved in plant biomass degradation. Summary table listing the numbers of CAZYme hits in each fungal genome in each of the CAZy categories corresponding to plant biomass degradation, organized by substrate.

**Additional file 24.** *Aspergillus* CK392 assembled contigs. *Aspergillus* CK392 genome.

**Additional file 25.** *Coniochaeta* CK134 assembled contigs. *Coniochaeta* CK134 genome.

**Additional file 26.** *Embellisia* CK46 assembled contigs. *Embellisia* CK46 genome.

**Additional file 27.** *Chaetomium* CK152 assembled contigs. *Chaetomium* CK152 genome.

**Additional file 28.** *Phoma* CK108 assembled contigs. *Phoma* CK108 genome.

**Additional file 29.** *Aspergillus* CK392 predicted coding sequences. *Aspergillus* CK392 predicted coding sequences.

**Additional file 30.** *Coniochaeta* CK134 predicted coding sequences. *Coniochaeta* CK134 predicted coding sequences.

**Additional file 31.** *Embellisia* CK46 predicted coding sequences. *Embellisia* CK46 predicted coding sequences.

**Additional file 32.** *Chaetomium* CK152 predicted coding sequences. *Chaetomium* CK152 predicted coding sequences.

**Additional file 33.** *Phoma* CK108 predicted coding sequences. *Phoma* CK108 predicted coding sequences.

## Data Availability

All data generated and analyzed during this study are included in this published article and its supplementary information files.

## Abbreviations

AT: Acyl transferase
C: Condensation
CAZyme(s): Carbohydrate active enzyme(s)
CBM: Carbohydrate-binding module
CDS: Coding sequences
CE: Carbohydrate esterase
DHN: 1,8-dihydroxynaphthalene
DMATS: Dimethylallyltryptophan synthase
DSE(s): Dark septate endophyte(s)
DSF: Dark septate fungi
FDR: False discovery rate
GH: Glycoside hydrolase
ITS: Internal transcribed spacer
KS: Beta-ketoacyl synthase
LPMO: Lytic polysaccharide mono-oxygenase
MEA: Malt extract agar
NRPS: Non-ribosomal peptide synthetase
PKS: Polyketide synthase
PL: Polysaccharide lyase
PSM(s): Identified peptide sequence(s)
SMIPS: Secondary Metabolites by InterProScan
SPOCS: Species Paralogy and Orthology Clique Solver
SSP(s): Small secreted protein(s)
TCA: Trichloroacetic acid
