# Supplementary figures and images for: Genomes and secretomes of Ascomycota fungi reveal diverse functions in plant biomass decomposition and pathogenesis
Source: BMC Genomics. 2019 Dec 12;20:976. doi: 10.1186/s12864-019-6358-x (PMC6909477; doi:10.1186/s12864-019-6358-x)

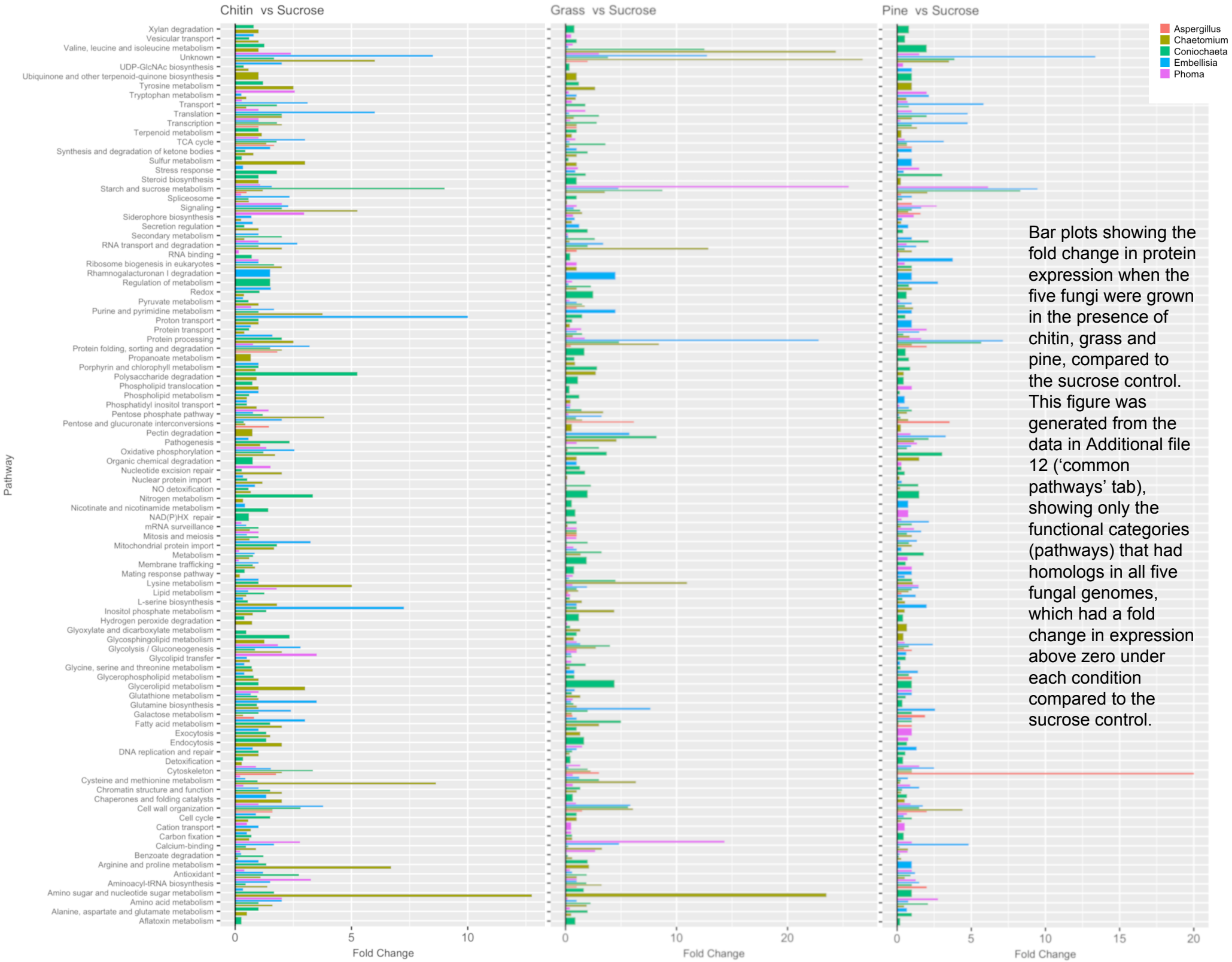

Supplement: Supplementary file 13 — Additional file 13. Fold change in expression of proteins, grouped by pathways. Barchart showing expression changes in each fungus, under each culture condition, grouped by metabolic pathways and other functional categories. The data for this Figure are provided in Additional file 12 ‘common pathways’ tab. [file 12864_2019_6358_MOESM13_ESM.pdf]
