# Supplementary material for: Genomes and secretomes of Ascomycota fungi reveal diverse functions in plant biomass decomposition and pathogenesis
Source: BMC Genomics. 2019 Dec 12;20:976. doi: 10.1186/s12864-019-6358-x (PMC6909477; doi:10.1186/s12864-019-6358-x)

Heatmap showing the expression levels of proteins with annotated functions in pathways for plant biomass degradation, defense and virulence (pathogenesis). Total protein counts in each replicate for each treatment condition are shown for each fungus. The coding sequence identifiers in the row labels to the right of the heatmaps are from the *Chaetomium* CK152 genome. The numbers of proteins are indicated by the color scale from dark blue to cyan to green, yellow, orange and red, increasing from bottom to top of the legend. The data used to generate this figure are presented in Additional file 14.

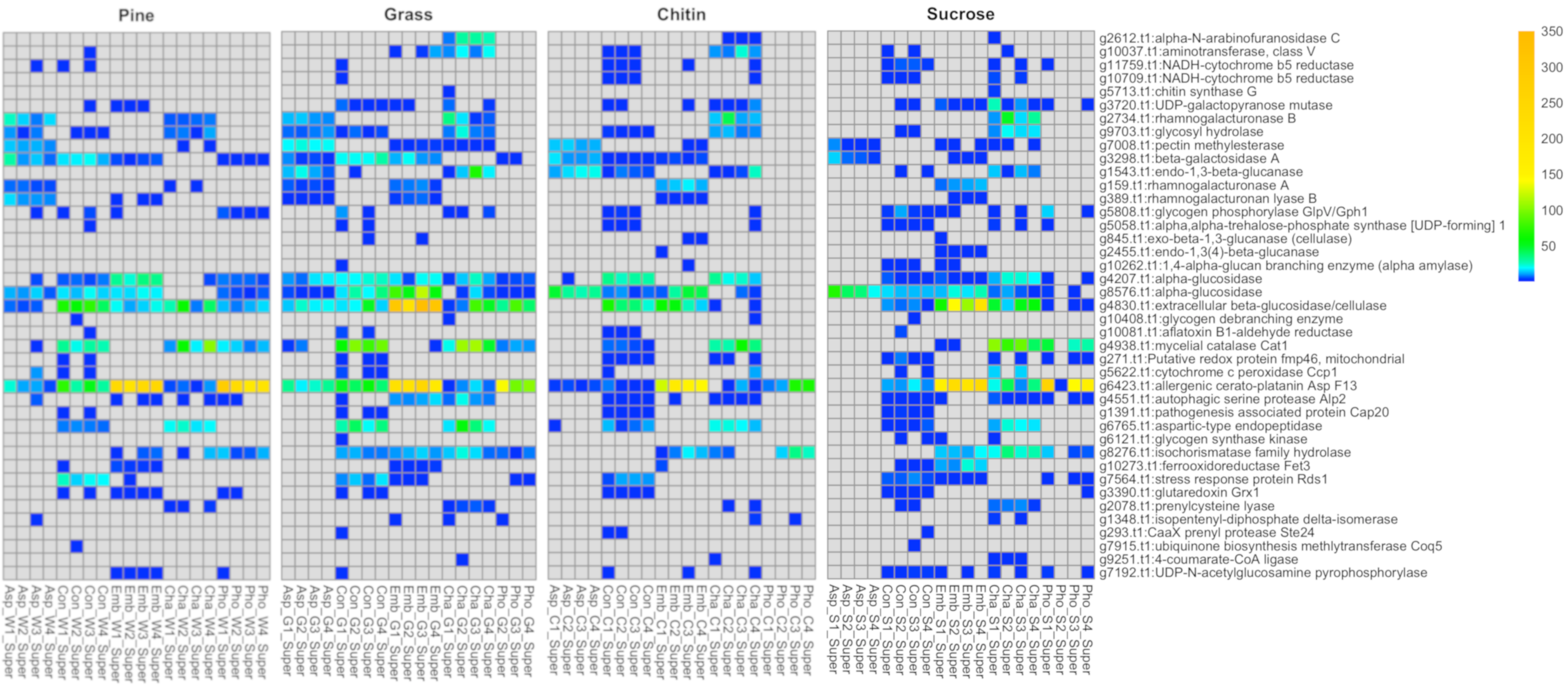

Supplement: Supplementary file 15 — Additional file 15. Heatmaps all replicates. Heatmap showing the expression levels of proteins with annotated functions in pathways for plant biomass degradation, defense and virulence (pathogenesis). Total protein counts in all replicates for each treatment condition are shown for each fungus. [file 12864_2019_6358_MOESM15_ESM.pdf]
