# Supplementary material for: Genomes and secretomes of Ascomycota fungi reveal diverse functions in plant biomass decomposition and pathogenesis
Source: BMC Genomics. 2019 Dec 12;20:976. doi: 10.1186/s12864-019-6358-x (PMC6909477; doi:10.1186/s12864-019-6358-x)
Supplement: Supplementary file 4 — Additional file 4. Labeled volcano plot chitin vs sucrose all fungi. Labeled volcano plot showing proteins with increased expression in all fungi in chitin cultures compared to sucrose control cultures. [file 12864_2019_6358_MOESM4_ESM.pdf]

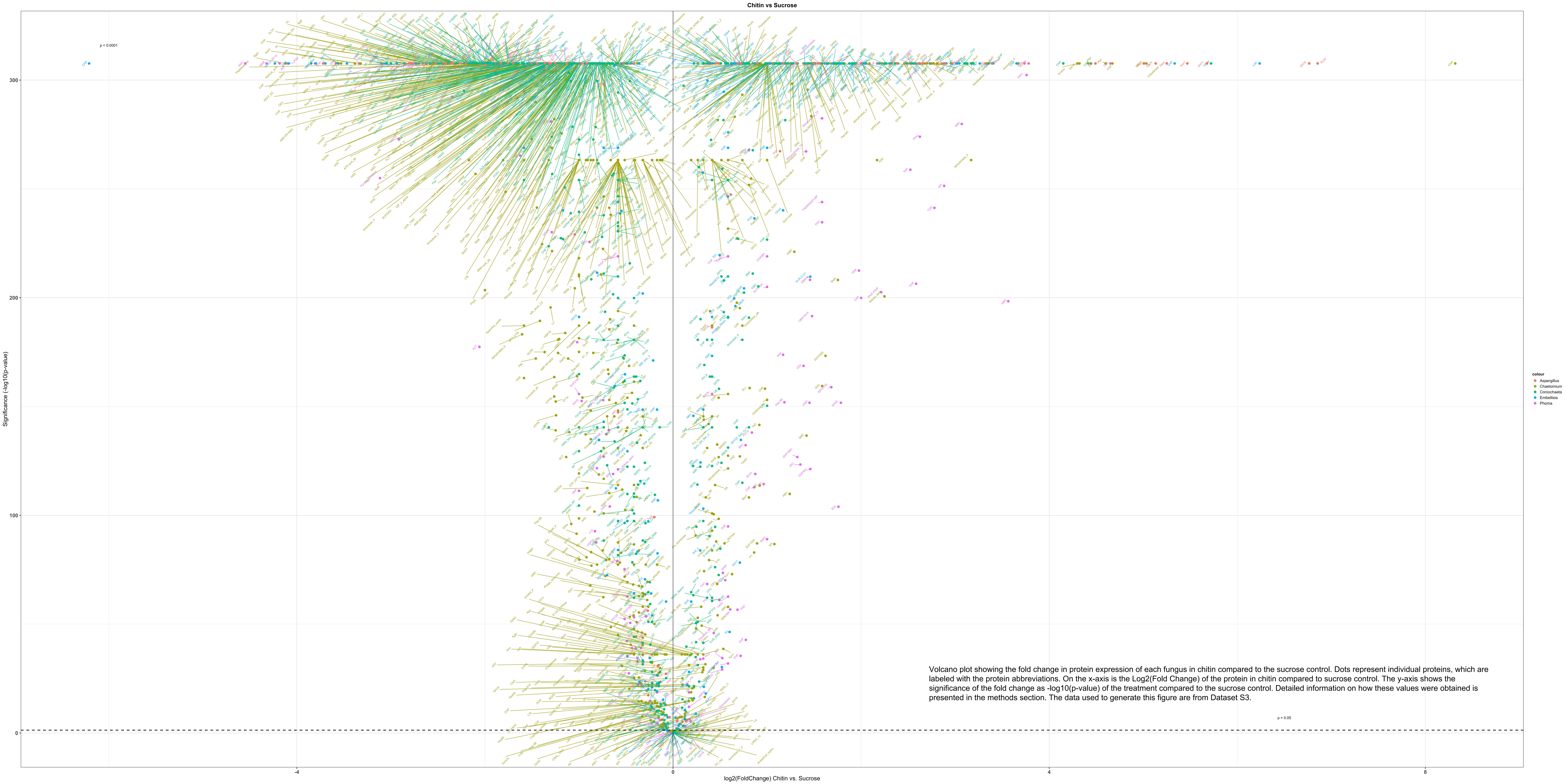

Volcano plot showing the fold change in protein expression of each fungus in chitin compared to the sucrose control. Dots represent individual proteins, which are labeled with the protein abbreviations. On the x-axis is the Log2(Fold Change) of the protein in chitin compared to sucrose control. The y-axis shows the significance of the fold change as -log10(p-value) of the treatment compared to the sucrose control. Detailed information on how these values were obtained is presented in the methods section. The data used to generate this figure are from Dataset S3.
