# Supplementary material for: Genomes and secretomes of Ascomycota fungi reveal diverse functions in plant biomass decomposition and pathogenesis
Source: BMC Genomics. 2019 Dec 12;20:976. doi: 10.1186/s12864-019-6358-x (PMC6909477; doi:10.1186/s12864-019-6358-x)
Supplement: Supplementary file 8 — Additional file 8. Labeled volcano plot Coniochaeta CK134 all culture conditions. Labeled volcano plot showing proteins with increased expression in Coniochaeta CK134 in chitin, grass and pine cultures compared to sucrose control. [file 12864_2019_6358_MOESM8_ESM.pdf]

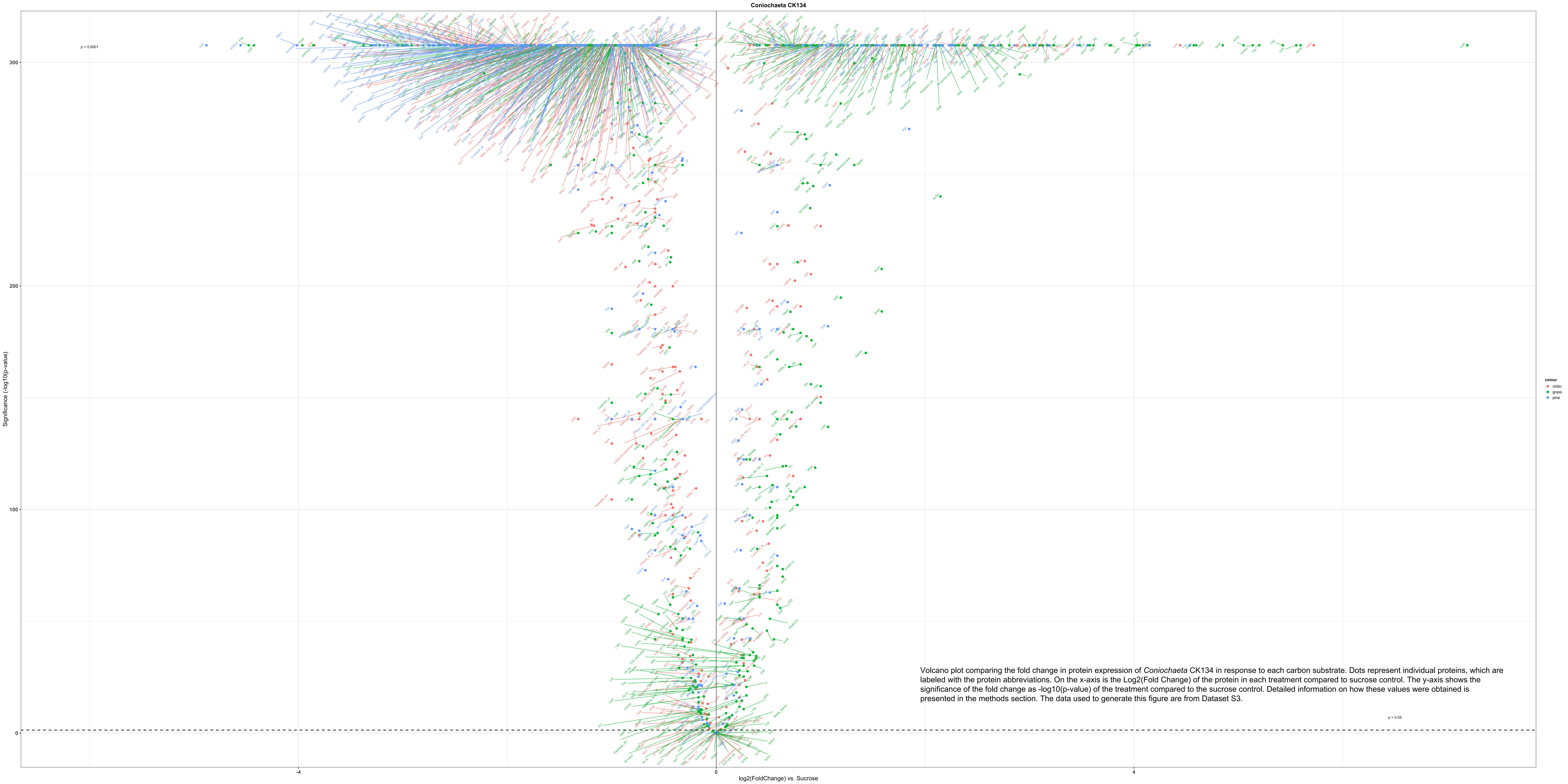


Volcano plot comparing the fold change in protein expression of *Coniochaeta* CK134 in response to each carbon substrate. Dots represent individual proteins, which are labeled with the protein abbreviations. On the x-axis is the Log2(Fold Change) of the protein in each treatment compared to sucrose control. The y-axis shows the significance of the fold change as -log10(p-value) of the treatment compared to the sucrose control. Detailed information on how these values were obtained is presented in the methods section. The data used to generate this figure are from Dataset S3.
